# Detection of genomic loci associated with chromosomal recombination using high-density linkage mapping in Setaria

**DOI:** 10.1038/s41598-017-15576-2

**Published:** 2017-11-09

**Authors:** Guanqing Jia, Haigang Wang, Sha Tang, Hui Zhi, Sichen Liu, Qifen Wen, Zhijun Qiao, Xianmin Diao

**Affiliations:** 1 Institute of Crop Sciences, Chinese Academy of Agricultural Sciences, Beijing, 100081 P.R. China; 2 Institute of Crop Germplasm Resources, Shanxi Academy of Agricultural Sciences, Taiyuan, 030031 People’s Republic of China

## Abstract

Meiotic recombination is essential to sexual reproduction and the generation of genetic diversity. Variation in recombination rates is presently of particular interest due to efforts being made to increase the rate of genetic gain in agricultural crops by breaking up large linkage disequilibrium blocks containing both beneficial and detrimental alleles. Here, a high-density genetic linkage map of Setaria was constructed using tunable genotyping by sequencing (tGBS) analysis of a population of recombinant inbred lines (RILs). Several regions of the Setaria genome exhibited significant levels of segregation distortion (SD), and recombination crossovers (COs) were also detected. The regions with high SD generally tended to have fewer COs, particularly for pericentromeric chromosomal areas. Recombination crossovers detected in Setaria were unevenly distributed across the genome and occurred more often in intergenic regions. Quantitative trait loci (QTLs) contributing towards the recombination frequency (Type I) and occurrence of COs in designated loci (Type II) were identified, and Type II QTLs garnered higher statistical power. The result of this study suggest that QTLs analysis of Type II traits using RILs might provide an opportunity to further understand meiotic recombination using high throughput genome sequencing and genotyping technologies.

## Introduction

Recombination (or chromosome crossover) plays a vital role in DNA damage repair, chromosome segregation, and the creation of novel haplotypes^1^, all of which contribute towards genetic diversity, allele assortment under natural selection^2^, and eukaryotic genome evolution^3^. Chromosomal double-strand breaks (DSBs) that occur during meiosis can be classified as crossovers (COs) or non-crossovers (NCOs), and lead to genomic exchange and non-exchange with synthesis-based strand annealing, respectively^4^. The rate and distribution of COs across the whole genome largely determines allele combinations and haplotype structure in the progeny of a segregating cross, and exhibits notable intraspecific^5^ and interspecific^6^ variation.

The pyramiding of favorable alleles in a single line is a primary objective in the selection breeding of several crop species, but is time consuming and restricted by the frequency of genetic recombination events. In maize, about 30–50 recombination events were expected in offspring derived from two parental hybrids^7,8^, and chromosomal COs were concentrated in the 5′ end of transcript^9^. Genetic factors including Rad51^10^, Ku70^11^, Rad50^12^, PHS1^13^ etc., have been found to contribute towards recombination events in plant and mammalian cells. Many quantitative trait loci (QTLs) contributing towards recombination frequency in plants have been identified using recombinant inbred lines (RILs) in recent years^14,15^. However, the genetic basis of the recombination events is presently still not clearly understood owing to the limited number of cloned genes and low statistical significance of detected QTLs^8^.

Foxtail millet (*Setaria italica* L.) is a globally cultivated cereal crop that was originally domesticated in China 11,500 years ago^16,17^. Recently, foxtail millet and its wild ancestor green foxtail (*Setaria viridis* L.) have been developed as novel models (named Setaria) for deciphering biological processes including C_4_ photosynthesis^18,19^, stress tolerance^20^, plant architecture^21^ and panicle development^22^. The benefits of using Setaria as a model species include its small genome, reproductive convenience, abundant genetic variation, and the availability of multiple reference genomes^23–26^. Numerous linkage maps have been constructed for foxtail millet using RFLP^27,28^, InDel^24^, SSR^29–31^ and SNP^23,32^ markers, and genomic features including segregation distortion (SD) and chromosome translocation have been identified in individual crosses. However, the low density of molecular markers used for linkage analysis in Setaria remains an obstacle for further genetic analysis of this emerging model.

In this trial, a RIL population of foxtail millet was genotyped using tunable genotyping by sequencing (tGBS) and a high-density linkage map was constructed. Genomic features including SDs and recombination event distributions were characterized and QTLs contributing towards variation in overall recombination frequency (Type I) as well as the frequency of COs at specific genomic loci (Type II) were systemically analyzed. The results of this study may provide an opportunity to identify the genomic regions involved in modulating the frequency of recombination events in plants.

## Results

### Construction of genetic linkage groups

**Figure 1:**
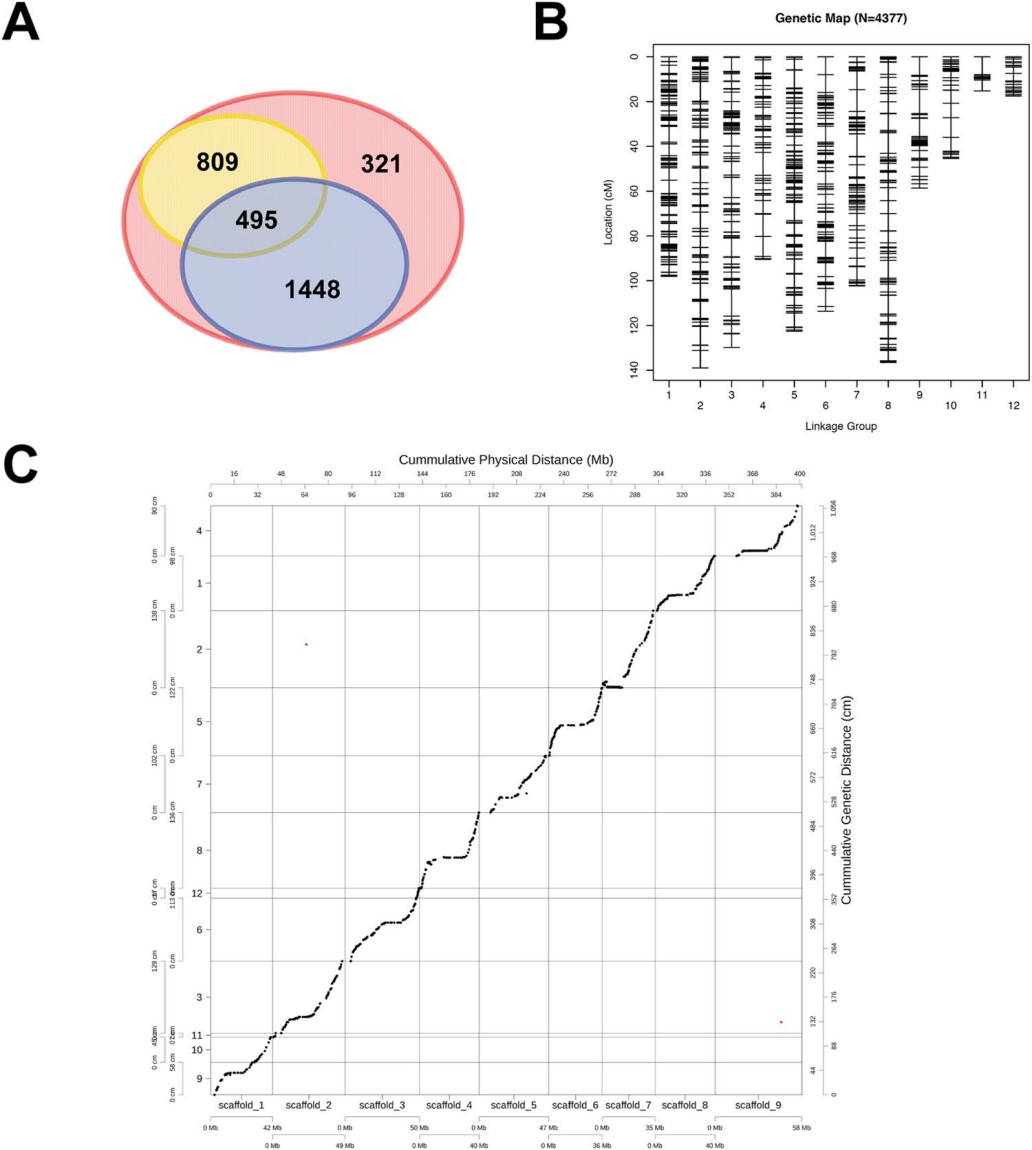
Characteristics and validation of the Setaria genetic map. (**A**) Venn diagram indicating the number of markers with significant SD (blue circle); number of marker pairs related to recombination loci (yellow circle); and total markers used for map construction (red circle). (**B**) Linkage groups constructed using SNP markers. (**C**) Comparisons between genetic linkage groups and the genome of “Yugu1”. The markers with consistent genetic and physical positions are indicated in black, while the markers with inconsistent genetic and physical positions are shown in red.

A total of 4,377 SNP markers (Fig. 1A) were used for constructing the linkage map employed here, of which 1,943 displayed significant segregation distortion (<0.05). A total of 1,304 recombination events were detected, of which 495 recombination loci were involved in segregation distortion. Twelve linkage groups with a total genetic distance of 1,069 cM were finally constructed, with an average spacing of 0.24 cM and maximum spacing of 12.1 cM between adjacent markers (Fig. 1B). Comparisons between individual linkage groups and their corresponding chromosomes revealed that nearly all SNP (99.9%) markers were ordered according to the reference genome sequence of “Yugu1” with high coverage (Fig. 1C), except for two loci mapped to chromosomes 2 and 9.

### Segregation distortion identified in the RIL population

**Figure 2:**
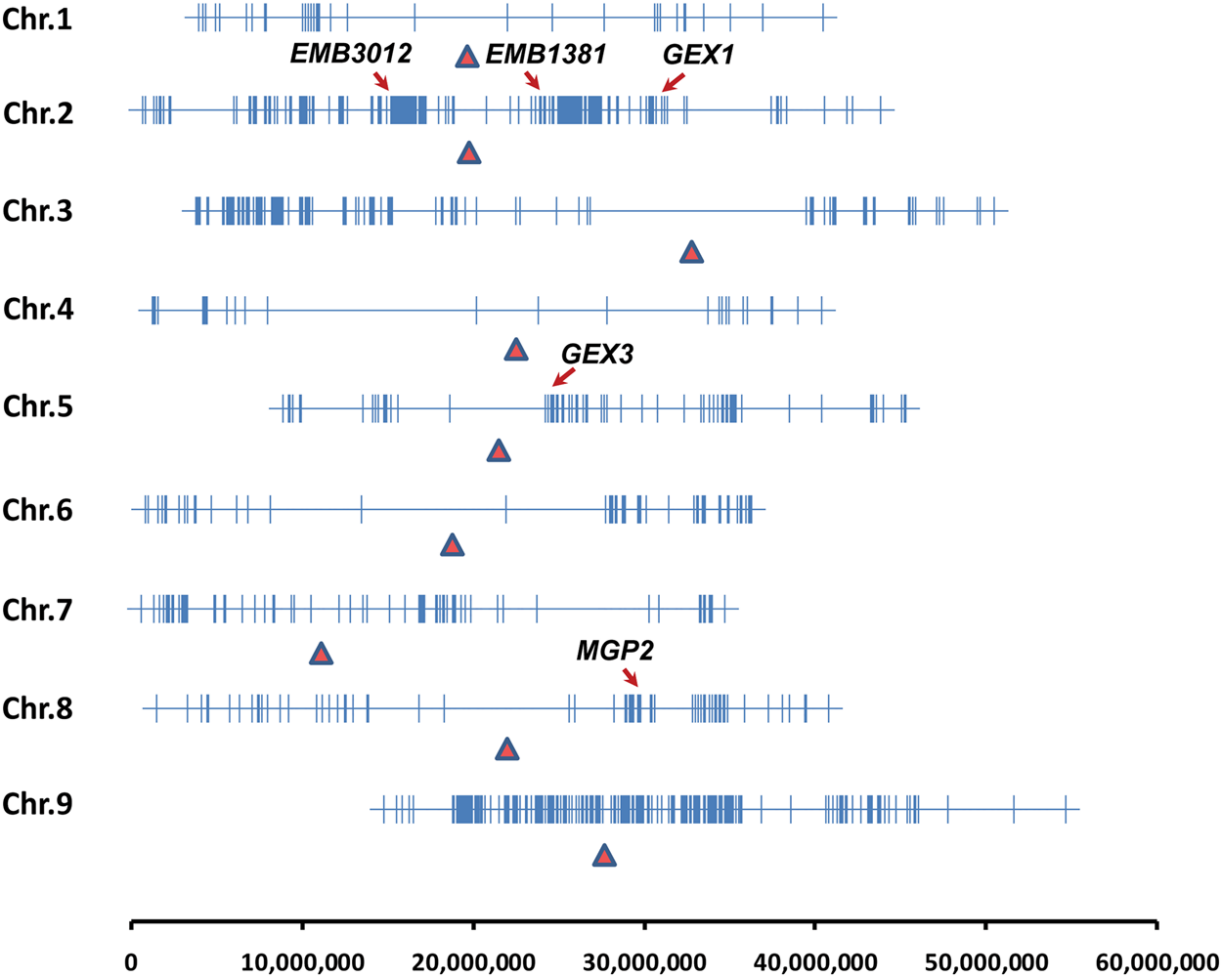
Distribution of SNPs exhibiting significant SD across the foxtail millet genome. Vertical lines mark the position of the SNP markers; the solid red triangles mark the centromeres; and the positions of specific genes of interest that overlap with regions of SD are marked by red arrows.

Given that the distorted segregation of SNP markers was observed in this trial (Fig. 1A), the distributions of markers exhibiting SD across all nine chromosomes were analyzed (Fig. 2). More SNP markers exhibiting significantly distorted segregation were mapped to chromosomes 2 and 9, and several genes including *EMB3012*, *EMB1381, GEX1, GEX3*, and *MGP2* that control embryo development, pollen development, and pollen germination were co-localized with groups of markers with distorted segregation. These observations suggested that seed developmental defectiveness might contribute towards gamete SD in the RIL population.

### Detection of whole-genome recombination events

**Figure 3:**
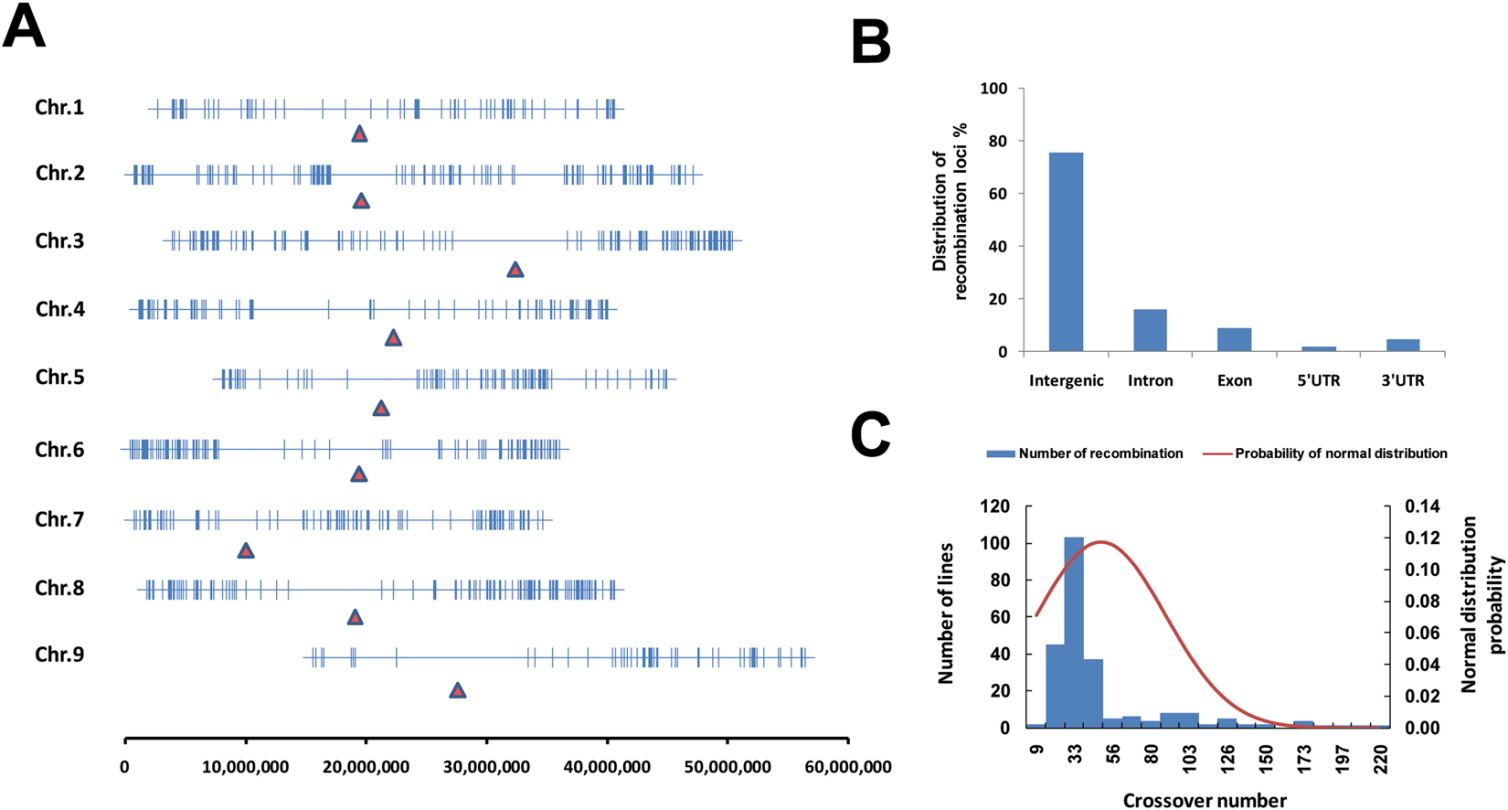
Recombination events detected in RILs. (**A**) Distribution of COs across foxtail millet genome. Positions of the COs are represented by vertical lines, while solid red triangles show the position of the centromeres. **(B)** Location of the COs relative to the annotated gene models in foxtail millet. **(C)** Distribution of the number of COs per RIL.

A total of 1,304 recombination events were detected in this trial (Fig. 1A). Chromosome crossover occurred most frequently in the gene rich euchromatic arms of each chromosome, while no recombination events were identified in the pericentromeric regions of each chromosome (Fig. 3A). Further CO events were detected in the intergenic regions, followed by the intron, exon, 3′UTR, and 5′UTR genomic areas (Fig. 3B).

The chromosomal recombination events observed per RIL in this study revealed that the crossover number detected in each segregating line was abnormally distributed (Fig. 3C), with an average of 49 recombination events observed per RIL. In more than half of the RILs, the observed number of crossovers ranged from 20 to 44. However, across the entire population, this number varied substantially from 9 to 220.

### Identification of Quantitative trait loci (QTLs) controlling overall recombination frequency (Type I) and recombination frequency at specific genomic loci (Type II)

**Figure 4:**
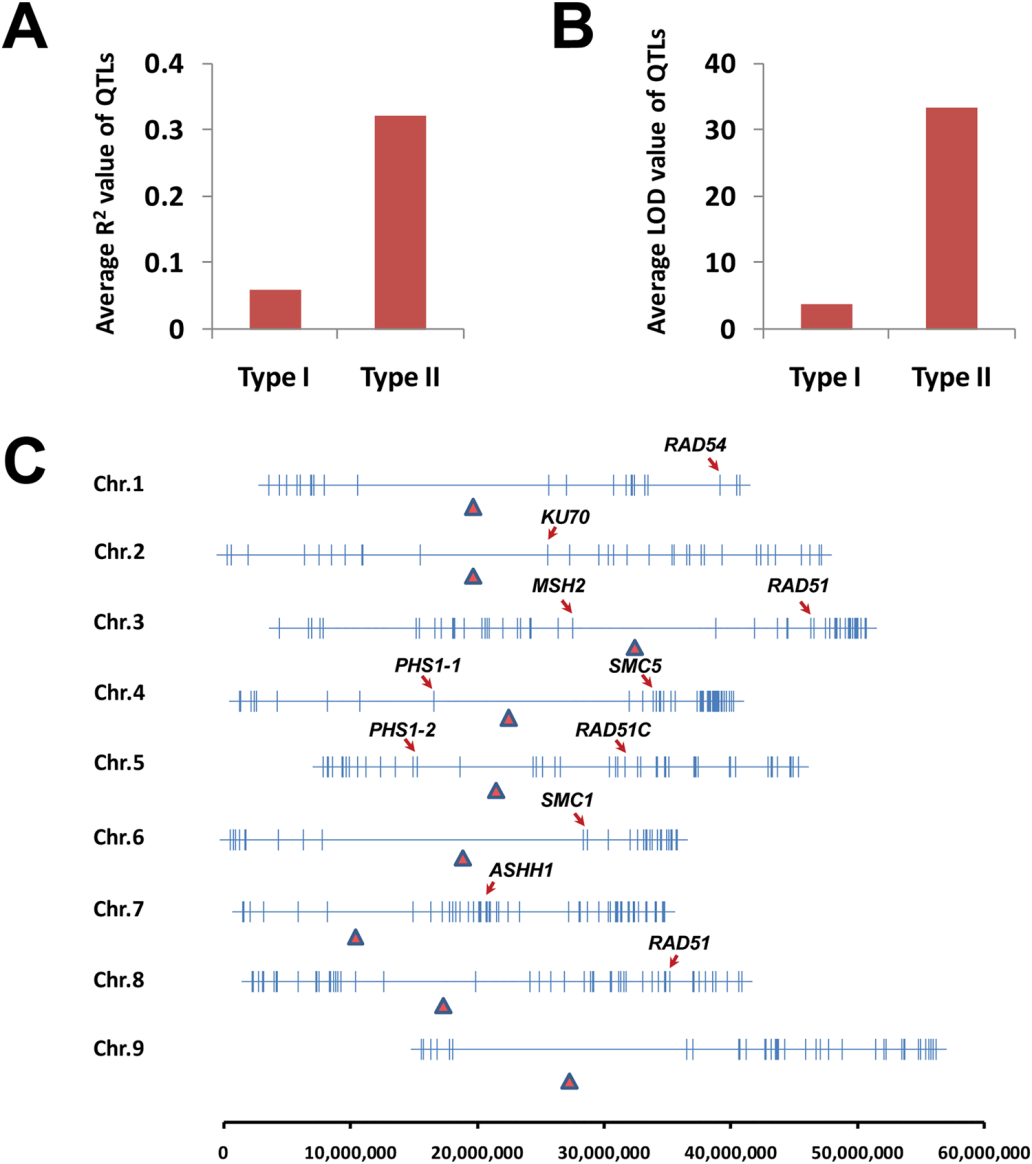
QTLs contributing towards recombination events in foxtail millet. (**A**) Average R^2^ value and (**B**) LOD value comparisons between Type I and Type II trait QTLs. (**C**) Distribution of QTLs detected for Type II traits across the foxtail millet genome. Peak positions of QTLs are marked by vertical lines; solid red triangles indicate the centromere positions; and the positions of particular genes of interest that overlap with QTL are marked by red arrowheads.

The 914 crossover loci identified in each recombined line were scored for QTL mapping analysis. A total of 617 QTLs (Table S1), including 3 *cis*- and 614 *trans*- loci, were identified to control the recombination (Type II) occurring in 341 crossover loci. Notably, in 180 of the genomic recombination loci, the crossovers in each locus were controlled by a QTL, with an average R^2^ value of 0.32 (ranging from 0.02 to 0.87; Fig. 4A). In terms of the recombination frequency of each line (Type I), three QTLs were identified on chromosome 8, with an average R^2^ value of 0.06 (Table 1; Fig. 4A and B). QTLs detected in this trial were distributed across all nine chromosomes. Several genes including *SMC5*, *SMC1*, *RAD51*, *KU70*, and *ASHH1* that are known to contribute towards recombination, chromosome segregation, and DNA repair were co-localized with QTLs determined to be contributors towards recombination occurrences in target genomic loci (Fig. 4C).

**Table 1:** QTLs detected that contribute towards the total number of recombination events (Type I) in the RILs.

QTL name	Chr.	Left marker (bp)	Right marker (bp)	LOD	A	R^2^
qrf1	8	7,085,610	7,222,415	3.6	9.42	0.06
qrf2	8	9,120,263	11,153,920	4.08	9.67	0.06
qrf3	8	12,436,515	23,848,966	3.93	9.42	0.06

## Discussion

In this trial, a high density genetic linkage map consisting of 4,377 SNP markers was constructed using tGBS analysis of a RIL population derived from a cross between “Yugu1”, an elite Setaria line, and “Hongmiaodabaigu”, a Setaria landrace. The density of genetic markers was significantly higher than previous reports of linkage maps constructed in Setaria^23,24,27–32^, suggesting that tGBS is effective for increasing marker density in linkage maps of Setaria, and is consistent with the findings in maize^8,33^ and other cereal crops. These types of linkage maps could provide an essential tool for dissecting genomic features in Setaria, an emerging model for deciphering the genetic basis of C_4_ photosynthesis^18^, plant and panicle architecture^34^ and water/nutrition usage^19,20^ in cereal crop species. The linkage map constructed in this trial includes markers with approximately 44% segregation distortion (Table S2), which were eliminated in skeleton maps constructed in a previous report^32^. This might explain why the map used in this study is denser than those reported previously.

The distorted segregation of molecular markers is common in segregating populations, and can be attributed to gametic and zygotic selection influenced by physiological or genetic factors^35^. In Setaria, the segregation distortion of markers has also been observed in many segregating populations^23,24,29–32^. In this trial, segregation distortion was observed in 44% of the SNP markers used for linkage analysis, which is consistent with results derived from maize studies using both CN-NAM and US-NAM populations characterized by GBS approaches^8^. The conclusions of this study suggest that segregation distortion does not affect molecular marker order determination in Setaria, which agrees with previous investigations^23^. Furthermore, the same conclusions have also been reported in other gramineous species, including maize^36^, as well as in maximum-likelihood models construction studies^37^. The distorted markers identified in this trial were unevenly distributed across all nine chromosomes of foxtail millet, while limited regions overlapped with known genes related to gametic and zygotic selection. These observations suggest that further research is essential to dissect the mechanisms and processes of genomic SD in Setaria. Interestingly, the markers with distorted segregation clustered in genomic regions where crossover events are rarely observed (Figs 3 and 4A), especially in the pericentromeric area, indicating that SD might affect the occurrence of recombination in foxtail millet; a finding also observed in maize^8^. Another possible explanation for this is that large linkage disequilibrium (LD) blocks with no recombination are more likely to harbor groups of deleterious alleles than areas of high recombination, where it is easy to select against deleterious alleles during long-term cultivation and breeding processes in Setaria. However, this conjecture requires further evaluation.

In previous work, recombination events were quantified by re-sequencing the recombined gametes sourced from meiosis, such as in humans^38–40^, yeast^41,42^, Arabidopsis^43,44^ and maize^9^. Chromosomal crossover events can be scored by counting the breakpoints between the stretches of marker alleles in segregating lines derived from the same F_1_ hybrid^8,14,15^. In this study, the second approach was applied in the dissection of the recombining features of the foxtail millet genome. We discovered that the majority of crossover events occurred in the intergenic regions, which is similar to the pattern observed in humans^39,40,45^. Analysis of the number of crossovers also suggested that there were 20–44 recombination events for most of the RILs in foxtail millet, which is in line with the 30–50 crossover events detected in maize lines derived from two parents via continuous selfing^7^. Our results suggest that genotyping a wider range of Setaria RIL populations in the future could be used to further explore the map loci responsible for the within-species variation in recombination frequency, as well as to investigate the mechanisms responsible for recombination hotspots in this species. Moreover, the genotyping of individual Setaria microgametophytes (i.e., pollen) from F_1_ individuals may elucidate the areas of segregation distortion that arise from pre-fertilization selection, and those that arise from selection during or after fertilization.

Recombinant inbred lines can be used to identify QTLs that contribute towards recombination events^8,14^. This is because over the course of single-seed descent, the genetic differences that impact recombination frequency segregate and become fixed in RILs. This differs from F_2_ individuals where all recombination events observed during meiosis in the F_1_ parent contain the same consistent set of recombination factors^14^. In this trial, the recombination frequency of each RIL was used as a trait (Type I) for QTLs analysis. Three QTLs were detected with relatively low statistical power, and similar results have also been observed in maize^8,14,15^. This might be explained by the rapid reduction in scorable recombination events during successive self-pollinating in RILs^5^, or parental differences in recombination frequency not being obviously reflected in the number of recombination events occurring in the RILs. Additionally, QTLs that control the occurrence of crossover events at specific loci (Type II) across the population were also tested in this trial. Surprisingly, a few QTLs that contribute to crossover events in many genome loci were identified with high statistical power, and some of the QTL hotspots may overlap with known genes related to meiosis in higher plants (Fig. 4C). This result suggests that the differences in recombination frequency between parental lines might be reflected in the occurrences of crossover events in a few diverse genomic regions in Setaria.

According to studies conducted in Arabidopsis, variations in the distribution of crossover events are controlled by both *cis* and *trans* genetic factors^1^. In this trial, most of the identified Type II QTLs were trans-acting factors (Fig. S1), which is consistent with reported results in maize^8^. It is possible that trans-acting proteins might play more exclusive roles in controlling recombination events in panicoid grasses. Given the high statistical power of Type II QTLs that control the occurrence of designated genomic loci, it can be inferred that the cloning or pyramiding of Type II QTLs controlling the recombination of target loci would aid precision molecular designing of crop cultivars in the future by increasing the recombination rates in regions recalcitrant to recombination, enabling an increase in the rate of genetic gain by breaking up positive and deleterious alleles that are currently tightly linked.

## Materials and Methods

### Recombinant inbred lines (RILs) population construction

The foxtail millet cultivar “Yugu1” and the landrace “Hongmiaodabaigu” (collected from Shanxi province of China) were used as parental lines. Hybridization was conducted according to the protocol described previously^27^. Cultivar “Yugu1” was pollinated using pollen grains collected from “Hongmiaodabaigu” and F_1_ hybrids were screened in the field based on morphological markers from the male parent “Hongmiaodabaigu”, and later verified using SSRs. A single validated F_1_ individual was bagged to produce an F_2_ population consisting of 288 plants. Each F_2_ plant was self-pollinated, and progenies of each F_2_ plant were advanced independently by single seed descent to generate a single RIL. The samples used in this study were from the lines advanced to the F_8_ generation.

### Genotyping of RILs

Fresh leaf tissue was collected from each RIL in the field during the foxtail millet growth period in Beijing in 2014. High quality DNA was extracted using the CTAB method^46^, and the samples were genotyped using tGBS^47^. Version 2.2 of the genome sequence of “Yugu1” as downloaded from Phytozome 12^23^ was used as a reference for the alignment of reads and SNP discovery.

### Construction of genetic linkage maps

A total of 4,680 Low Missing Data (LMD; missing data rate <30%) filtered among the 236 RILs (individuals that were heterozygous for >5% of SNP sites were removed) were used for constructing the genetic maps. The grouping, ordering, and genetic distance between the linked markers were inferred using the R/qtl software package^48^, using the following parameters: rf = 0.25, lod = 16, and minimum SNPs of each LinkageGroup (LG) = 50. All SNPs genotyped as heterozygous were treated as missing values in subsequent analyses.

### Segregation distortion analysis

The proportion of “Yugu1” or “Hongmiaodabaigu” alleles at each locus were tested for SD against the 1:1 segregation ratio expected of a RIL population using a chi-square test. SNP markers exhibiting SD (p < 0.05) were scored as SD markers.

### Detection of recombination events

Recombination events were scored by counting the breakpoints between stretches of marker alleles from one parent to another in the RIL mapping data according to the marker positions ordered by linkage analysis. The total number of recombination events summarized in each RIL was used as a quantitative trait. The location of COs was considered to be the mid-point between the physical positions of the two flanking SNP markers. Genome annotations of all COs were retrieved from Phytozome 12 (https://phytozome.jgi.doe.gov/jbrowse/index.html?data=genomes%2FSitalica/).

### QTLs identification

The genetic map, consisting of 4,377 SNP markers, was used for QTLs analysis using composite interval mapping (CIM) and the standard model (Model 6) as implemented by Windows QTL Cartographer V. 2.5^49^, with a walk speed of 1.0 cM and window size of 10.0 cM. An LOD score of 3.0 was used as the significance threshold. The number of recombination events observed in each RIL was used as the phenotype for mapping Type I QTL. For type II QTL, QTLs controlling recombination at each CO locus were mapped separately. RILs with a CO at a given location were scored as “2” and RILs without a crossover at the location were scored as “1”. Only COs that occurred in more than two different lines were selected for Type II QTL mapping analysis.

### Data availability

All QTL data are available in the manuscript.

## Electronic supplementary material

Supplementary file
