# Supplementary material for: Detection of genomic loci associated with chromosomal recombination using high-density linkage mapping in Setaria
Source: Sci Rep. 2017 Nov 9;7:15180. doi: 10.1038/s41598-017-15576-2 (PMC5680217; doi:10.1038/s41598-017-15576-2)
Supplement: Supplementary file 1 — Supplementary file [file 41598_2017_15576_MOESM1_ESM.pdf]

Detection of genomic loci associated with chromosomal recombination using high-density linkage mapping in Setaria

Guanqing Jia[1#], Haigang Wang[1#], Sha Tang[2], Hui Zhi[2], Sichen Liu[2], Qifen Wen[2], Zhijun Qiao[2], Xianmin Diao[1*]

1. Institute of Crop Sciences, Chinese Academy of Agricultural Sciences, Beijing, 100081, P.R. China;

2. Institute of Crop Germplasm Resources, Shanxi Academy of Agricultural Sciences, Taiyuan, 030031, People's Republic of China

#: These authors contributed equally to this work.

[*] Corresponding Author: Xianmin Diao

Supplementary information

Fig. S1. Genomic positions of the recombination loci and QTLs controlling the occurrence of COs in foxtail millet. The x-axis indicates the genomic distributions of the recombination loci, while the y-axis shows the genomic distributions of QTLs detected in this trial. The red dashed line signifies the co-localizing positions of the genomic breakpoint regions and QTLs identified in this trial. The nine Setaria chromosomes were arranged according to the order of the reference genome of "Yugu1", and the genomic position of each point was indicated using cumulative physical distance (bp) based on the reference genome sequence.

Table S1. Detailed information of QTLs controlling the recombination occurrences at specific genomic loci in Setaria.

Table S2. Segregation distortion (P<0.05) of SNPs detected in this trial.

Fig. S1

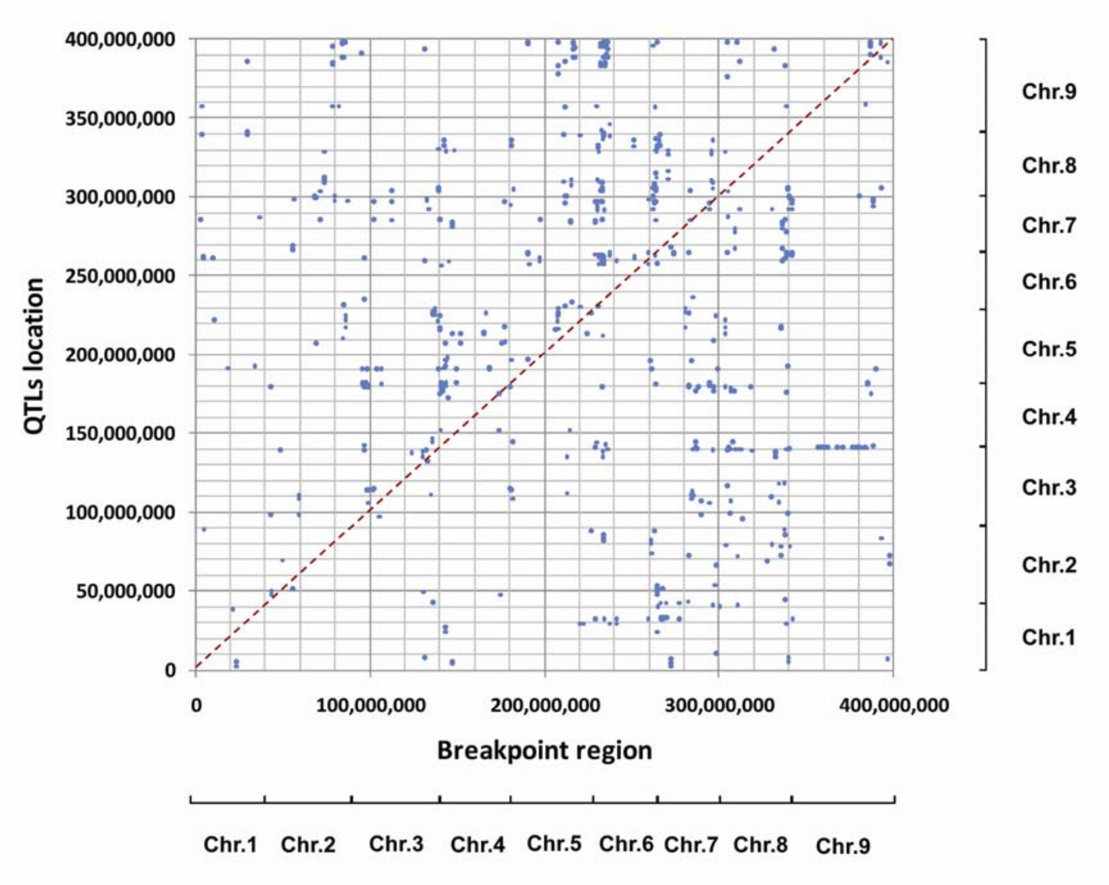

400,000,000
350,000,000
300,000,000
250,000,000
200,000,000
150,000,000
100,000,000
50,000,000
0
QTLs location
0
100,000,000
200,000,000
300,000,000
400,000,000
Breakpoint region
Chr.9
Chr.8
Chr.7
Chr.6
Chr.5
Chr.4
Chr.3
Chr.2
Chr.1
Chr.1 Chr.2 Chr.3 Chr.4 Chr.5 Chr.6 Chr.7 Chr.8 Chr.9

Table S1

Table S1. Detailed information of QTLs controlling the recombination occurrences at specific genomic loci in Setaria.

Trait(breakpoint region)		Chromosome	Left(bp)	Right(bp)	LOD	A	R^2
**Chr.1**	3,361,266	7	19,480,559	20,080,988	78.84	-0.45	0.73
	4,097,266	8	37,650,521	37,800,651	67.24	0.5	0.74
		9	15,651,385	18,576,632	77.7	0.5	0.74
	4,584,311	2	46,064,097	47,229,995	88.81	-0.48	0.69
	4,742,849	6	31,384,390	31,972,241	58.22	-0.5	0.6
		6	32,057,389	32,463,875	55.3	-0.5	0.6
	10,318,605	6	31,384,390	31,956,712	70.19	0.49	0.71
	11,116,049	5	39,176,254	40,145,371	136.74	0.48	0.73
	21,145,059	1	37,749,543	39,404,706	241.8	0.5	0.73
	19,327,361	5	9,319,145	9,913,514	19.77	0.47	0.62
	23,741,056	1	2,654,735	4,067,797	22.96	-0.47	0.43
		1	4,807,833	6,625,936	21.04	-0.49	0.62
	30,092,598	8	37,493,996	37,836,052	208.9	0.49	0.72
		8	39,659,931	40,513,595	213.78	0.49	0.72
		9	42,942,707	43,659,622	193.05	0.49	0.72
	33,651,516	5	9,913,514	11,092,224	53.95	0.5	0.76
		5	11,092,224	13,395,803	62.29	0.5	0.76
	37,307,241	7	21,813,287	23,290,452	47.98	0.48	0.54
**Chr.2**	910,747	2	5,922,737	6,625,955	139.85	-0.47	0.72
		2	7,020,810	8,760,425	134.38	-0.47	0.72
	1,138,086	3	6,741,112	7,686,927	3.75	0.04	0.07

	1,172,013	4	37,285,523	37,610,411	5.36	-0.05	0.1
		4	38,358,161	39,440,701	3.62	0.06	0.08
	6,795,603	3	47,608,081	48,086,069	55.58	-0.44	0.63
	7,272,688	2	27,583,917	36,384,580	4.17	-0.07	0.07
	13,804,596	2	9,020,619	11,279,783	62.61	-0.47	0.56
		7	614,261	2,012,894	90.78	0.49	0.72
		7	2,888,596	14,750,555	85.22	0.49	0.72
	14,856,822	7	33,120,971	34,235,173	182.65	0.49	0.85
	16,641,239	3	6,409,016	7,686,927	9.17	-0.12	0.22
		3	17,660,771	18,694,440	5.29	0.08	0.13
		3	18,830,392	21,148,824	6.77	0.08	0.11
	16,698,927	3	6,409,016	7,686,932	17.18	-0.25	0.45
		3	18,603,221	21,148,824	4.76	0.09	0.1
	26,814,638	7	33,501,487	34,235,172	9.26	0.2	0.23
		7	34,231,771	34,649,194	12.19	0.2	0.23
	27,631,076	5	24,374,189	24,771,351	6.38	0.08	0.11
	27,631,339	7	33,501,487	34,235,172	13.6	0.17	0.3
	29,739,649	7	19,480,559	20,080,988	92.3	0.49	0.73
		7	20,161,820	21,335,925	91.42	0.48	0.73
		8	2,249,714	3,666,110	81.4	0.48	0.73
	32,134,511	8	6,937,176	7,992,779	28.89	-0.47	0.55
		8	8,967,828	13,414,380	25.05	-0.46	0.55
		8	10,523,638	25,820,332	26.53	-0.47	0.55
		8	27,691,137	28,916,268	7.07	0.17	0.13
	36,614,723	9	41,216,640	42,942,707	169.01	-0.48	0.88

		9	52,759,823	54,197,810	146.64	-0.49	0.74
	36,867,201	9	15,651,385	18,576,632	71.16	-0.48	0.72
		9	43,431,434	43,659,622	46.95	-0.48	0.74
	37,268,102	7	32,172,306	32,749,614	15.53	-0.49	0.39
		7	34,235,172	34,649,194	17.37	0.28	0.38
	39,613,221	9	15,651,385	40,471,848	2.74	0.5	0.82
	41,978,983	5	28,446,893	33,120,715	4.12	-0.06	0.07
	42,432,657	9	45,631,606	47,380,798	63.86	0.47	0.69
		9	54,197,810	55,084,603	57.54	0.47	0.69
		9	55,084,603	56,221,686	119.65	0.47	0.71
	43,045,470	9	45,631,606	47,380,798	38.54	-0.48	0.61
	43,076,462	6	1,399,900	1,579,120	9.53	-0.16	0.28
	43,461,807	9	55,084,603	56,221,686	10.23	-0.24	0.07
	43,645,275	5	35,477,616	38,308,328	19.71	-0.46	0.35
		5	39,176,254	40,974,847	9.18	-0.12	0.35
		5	42,003,845	42,996,909	10.35	-0.16	0.26
	45,740,668	7	32,172,306	32,749,614	51.15	0.36	0.42
**Chr.3**	3,742,226	5	9,319,145	9,755,134	4.04	0.08	0.15
	3,813,842	4	38,817,105	39,440,701	3.68	0.07	0.12
		4	39,449,540	39,738,400	4.6	-0.04	0.08
		4	39,790,677	40,048,323	6.1	-0.05	0.1
		5	7,698,529	9,462,158	7.9	0.07	0.12
	4,105,674	9	48,540,183	55,084,603	4.32	-0.24	0.07
	5,392,336	4	37,285,523	37,610,411	61.21	-0.5	0.62
	5,411,617	4	38,383,279	38,570,143	3.25	-0.51	0.65

	5,589,692	4	37,285,523	37,610,411	71.26	-0.49	0.72
		6	31,384,390	31,961,872	37.75	-0.44	0.41
	5,691,213	4	37,139,248	37,285,523	6.7	0.12	0.11
		4	38,383,223	38,599,536	10.72	0.42	0.34
		6	5,033,933	7,270,709	4.9	-0.07	0.05
	6,041,631	3	47,358,386	48,086,097	13.89	0.51	0.26
		4	884,325	1,188,074	176.92	-0.49	0.78
	6,071,749	3	22,541,256	24,743,581	4.18	0.05	0.07
	6,253,757	4	37,610,411	38,806,496	12.43	0.21	0.18
		4	39,659,594	39,738,400	8.54	-0.15	0.07
		4	39,790,677	40,035,645	4.99	-0.15	0.12
	6,661,915	4	37,770,349	38,383,279	6.75	-0.14	0.18
		4	39,735,272	40,035,645	45.18	0.49	0.68
	6,692,156	4	38,383,279	38,806,496	14.75	0.21	0.09
		4	38,817,105	39,428,416	21.66	0.5	0.54
		4	39,428,416	39,449,540	17.92	0.3	0.29
		4	40,035,645	40,048,323	71.31	-0.5	0.84
	7,120,658	5	7,698,529	8,008,145	90.3	-0.32	0.37
	7,272,748	4	38,383,279	38,383,279	7.47	0.14	0.14
		5	7,698,529	8,008,145	5.91	-0.13	0.1
	7,438,219	5	7,698,529	8,008,145	9.85	0.34	0.13
	7,604,275	5	7,698,529	8,008,145	20.5	0.5	0.36
	8,214,701	3	15,091,423	17,660,771	154.21	0.49	0.78
	8,384,264	3	22,435,861	26,077,505	6.7	-0.09	0.12
		4	37,149,407	37,285,523	4.25	-0.03	0.06

		4	37,770,349	38,383,223	5.22	0.06	0.09
	10,125,413	3	22,435,861	24,743,581	82.93	0.5	0.77
	11,260,371	7	19,480,559	20,080,988	23.82	0.61	0.48
		7	31,154,026	31,147,307	8.78	0.39	0.49
	11,281,148	3	23,045,873	37,855,231	50.61	0.49	0.73
	12,706,384	5	7,698,529	8,008,145	4.73	-0.11	0.17
		5	8,136,067	8,688,052	3.55	-0.07	0.06
	14,254,813	3	6,347,743	6,680,278	24.87	-0.48	0.66
	14,907,744	4	38,711,133	39,440,701	4.81	-0.5	0.92
		4	38,711,133	39,440,701	8.08	-0.5	0.92
		5	7,698,529	8,008,145	4.22	-0.07	0.1
	21,324,930	7	20,031,936	21,757,983	13.2	0.51	0.34
		7	30,888,816	31,147,307	6.91	0.39	0.16
		8	1,907,189	3,438,310	58	-0.5	0.7
	32,093,652	3	45,727,922	46,168,027	13.11	-0.5	0.59
	38,398,359	3	43,125,846	45,092,476	20.87	0.49	0.64
		3	46,970,660	48,086,097	40.58	-0.49	0.68
	39,703,604	2	7,511,317	11,962,955	19.68	0.46	0.28
	40,521,313	6	29,290,131	31,124,408	64.01	0.49	0.65
		9	50,826,850	51,156,786	4.96	0.14	0.24
	40,794,890	7	32,172,306	32,849,878	222.2	-0.5	0.96
		7	32,849,878	33,344,431	226.8	-0.5	0.96
	40,653,999	1	7,349,135	7,725,173	82.16	-0.35	0.65
	41,484,715	3	47,358,386	48,086,069	42.67	-0.51	0.65
	42,314,793	3	40,874,235	44,696,787	3.87	0.07	0.07

	42,795,851	7	26,950,787	28,812,910	68	0.44	0.72
	43,296,878	3	20,050,828	21,148,824	6.96	-0.11	0.11
	44,017,659	4	2,093,701	4,074,903	5.16	0.08	0.1
		4	5,387,428	10,419,778	3.31	-0.06	0.05
	45,043,937	5	43,352,047	43,812,769	122.06	-0.47	0.71
		5	43,784,082	44,944,140	126.69	-0.49	0.71
		5	44,441,904	45,012,784	129.67	-0.49	0.71
	45,424,162	2	0	467,805	101.48	0.46	0.57
	45,518,885	5	43,352,047	43,812,769	122.06	-0.47	0.71
		5	43,352,047	43,812,769	126.69	-0.49	0.71
		5	44,441,904	45,012,784	129.67	-0.49	0.71
		6	283,715	543,648	31	0.46	0.41
	47,199,201	5	38,308,328	42,003,845	39.49	-0.44	0.51
	48,143,127	5	8,008,145	9,462,158	6.06	0.08	0.11
		8	1,907,189	2,249,636	4.15	0.09	0.11
		8	3,863,879	4,146,658	3.37	0.06	0.06
		8	29,284,440	30,565,945	2.83	-0.05	0.05
	48,707,284	4	10,454,724	20,407,071	4.16	0.1	0.07
		4	33,516,646	34,324,523	4.06	0.17	0.1
	48,795,959	4	37,139,135	37,610,411	51.41	-0.48	0.7
	48,932,303	5	33,611,551	34,578,543	5.13	0.11	0.09
		5	35,477,616	38,308,328	35.69	0.48	0.57
		5	42,003,845	43,712,480	41.04	0.48	0.57
	49,019,749	7	19,480,559	20,031,936	26.59	-0.3	0.54
		7	20,161,820	21,063,298	7.28	-0.15	0.27

		7	21,070,185	21,757,983	5.77	-0.13	0.24
	49,099,124	4	32,840,048	34,180,120	58.78	-0.5	0.73
	49,693,051	6	27,327,917	28,307,479	4.29	0.05	0.07
	50,086,240	4	37,285,523	37,610,411	4.59	0.08	0.06
		4	38,817,105	39,440,701	4.01	-0.11	0.19
		4	39,735,272	39,738,400	3.6	0.05	0.03
	50,237,269	4	37,139,099	37,149,407	9.96	-0.15	0.23
	50,240,791	4	34,180,120	35,775,576	21.5	0.21	0.34
**Chr.4**	1,031,512	8	33,988,459	35,379,819	37.43	0.49	0.61
	1,055,737	8	30,866,818	31,498,529	5.23	-0.11	0.18
	1,112,939	5	9,319,109	9,913,514	61.41	0.4	0.63
	1,219,888	4	38,383,279	38,817,105	4.06	0.05	0.07
		4	39,440,701	39,735,272	4.27	0.05	0.07
		4	39,738,400	40,035,645	3.19	0.05	0.06
	1,262,794	8	27,436,066	28,446,735	50.33	-0.48	0.69
	1,315,824	5	13,386,169	18,520,351	3.37	-0.11	0.07
		5	14,343,072	24,374,189	7.7	-0.09	0.13
	1,816,464	5	9,913,514	11,092,224	3.59	0.11	0.06
		5	14,343,072	14,789,074	3.66	-0.1	0.07
		5	24,440,238	24,771,351	3.67	-0.14	0.07
	1,816,480	1	24,430,388	26,421,404	95.46	-0.37	0.67
		1	26,421,404	27,140,873	93.1	-0.39	0.67
	2,178,948	5	14,789,074	24,440,251	3.91	0.08	0.07
	2,892,843	6	29,906,982	31,050,799	28.98	-0.48	0.6
	3,293,896	4	30,025,948	31,760,526	43.81	0.44	0.6

	5,411,253	5	30,702,027	31,414,384	5.22	-0.06	0.09
	5,583,909	1	3,980,264	4,677,865	32.24	0.49	0.65
		1	5,009,557	6,626,055	51.8	0.49	0.65
	5,545,537	7	15,615,857	17,013,722	176	0.49	0.64
		7	17,013,722	17,535,386	167.35	0.49	0.64
		7	17,675,369	18,134,160	174.1	0.49	0.64
	6,000,306	8	28,446,735	29,036,632	56.16	0.5	0.72
	8,016,885	4	39,790,677	40,048,312	3.48	-0.08	0.1
		5	7,698,529	9,462,158	3.94	0.07	0.09
	10,264,711	5	24,374,189	24,771,351	5.78	-0.07	0.11
		5	30,790,000	31,567,496	3.62	-0.51	0.96
	23,893,816	5	30,702,652	31,414,384	3.48	-0.04	0.06
		5	31,567,496	32,641,148	3.48	-0.05	0.05
	24,055,978	5	43,784,082	44,978,312	4.42	0.14	0.13
	26,749,490	5	8,008,145	8,115,317	9.05	0.5	0.98
		5	8,688,052	9,462,158	5.39	0.07	0.13
	32,300,287	4	10,592,021	31,760,526	3.75	-0.1	0.06
		4	32,840,048	33,516,646	3.7	-0.11	0.06
	33,187,874	2	5,922,737	6,625,955	45.39	-0.83	0.56
	33,920,584	5	24,440,238	26,552,245	9.09	0.17	0.16
	35,078,506	5	25,522,728	26,296,322	3.52	0.08	0.07
	35,463,370	7	30,660,022	30,660,086	48.54	0.36	0.13
	35,647,851	5	34,866,033	39,176,254	21.71	0.35	0.1
	38,370,720	7	29,547,829	30,409,270	3.02	-0.04	0.05
	38,584,840	3	23,052,378	24,743,581	14.38	0.11	0.32

	38,655,315	4	37,285,523	37,610,411	5.36	-0.06	0.1
		4	38,383,279	38,599,497	5.16	0.07	0.09
	39,736,819	5	14,343,072	14,014,955	5.02	-0.08	0.08
	39,764,484	8	30,866,818	31,498,529	7.06	-0.13	0.15
		8	33,988,492	35,570,051	4.95	0.23	0.15
	39,764,521	3	22,435,861	24,743,581	5.63	-0.06	0.09
	39,913,161	8	2,582,156	3,203,552	3.65	-0.11	0.06
	40,089,010	3	17,660,771	18,694,440	40.6	-0.49	0.64
		4	2,264,250	2,582,156	108.35	-0.46	0.78
**Chr.5**	8,648,755	6	33,973,839	34,544,886	4.12	0.11	0.07
		6	34,746,716	35,522,223	4.57	0.12	0.08
	8,401,684	5	14,335,952	24,440,251	4.3	-0.06	0.06
		9	54,197,810	55,084,603	79.23	0.49	0.66
		9	55,084,603	56,221,686	81.31	0.49	0.66
	9,390,651	6	28,307,479	31,972,241	10.98	-0.23	0.19
	14,553,870	6	29,906,982	31,050,799	3.75	-0.08	0.13
		6	31,384,390	31,972,241	3.1	-0.08	0.13
	15,898,973	7	19,480,559	20,031,948	63	-0.5	0.63
	24,331,406	5	33,325,336	34,578,543	3.7	0.05	0.07
	24,605,798	5	34,578,543	34,866,033	5.75	-0.11	0.1
		5	38,308,328	42,003,845	31.36	0.5	0.42
		5	42,003,845	42,996,909	13.26	0.49	0.18
	25,671,685	9	55,084,603	55,976,361	46.6	0.5	0.67
	25,972,612	5	34,385,309	34,578,543	15.21	-0.33	0.22
		5	43,352,047	44,441,904	51.67	0.49	0.58

		6	283,715	543,597	68.67	-0.5	0.76
	26,132,278	5	44,846,088	45,012,784	63.19	-0.49	0.65
		9	35,395,579	38,196,068	52.4	0.49	0.57
		9	40,248,082	40,697,539	53.23	0.49	0.57
	29,735,003	9	43,286,118	43,659,622	3.42	-0.09	0.05
	29,838,178	6	543,597	674,824	3.55	-0.07	0.08
	29,012,683	8	8,504,562	8,967,829	4.08	0.08	0.07
	29,012,694	8	37,896,389	39,659,931	5.18	-0.11	0.12
	30,035,361	7	30,258,612	30,592,996	92.41	0.46	0.67
		7	34,235,172	34,649,194	62.72	0.49	0.59
		9	14,616,159	18,576,632	7.1	0.08	0.11
	29,880,305	7	34,231,711	34,649,194	24.26	0.46	0.39
	30,136,434	7	34,231,711	34,649,194	3.9	0.08	0.13
	30,361,477	3	21,148,021	22,435,876	6.35	0.11	0.12
		3	43,125,875	45,346,672	3.02	0.06	0.05
	31,941,971	4	10,419,778	20,407,380	4.93	0.12	0.08
	32,517,328	8	5,577,905	7,992,779	4.86	0.14	0.09
		8	8,967,829	25,820,332	5.43	0.16	0.09
	32,877,407	7	18,028,639	18,431,429	4.33	-0.07	0.08
		7	18,566,681	19,125,603	3	-0.06	0.06
	33,846,061	6	3,445,153	4,020,085	5.66	0.07	0.11
	34,444,051	9	45,631,606	49,055,032	8.22	0.25	0.14
		9	51,156,786	54,043,426	4.05	0.08	0.07
		9	54,197,810	55,084,603	75.1	-0.46	0.57
		9	55,084,603	56,221,686	53.34	-0.45	0.55

	35,019,258	9	45,631,606	47,380,798	11.57	-0.47	0.34
		9	51,974,382	54,197,810	20.4	-0.46	0.34
	38,742,291	6	1,238,859	1,579,120	8.92	0.34	0.13
		8	38,182,222	38,766,407	3.45	-0.11	0.05
		1	29,672,513	31,490,543	4.37	0.16	0.07
	39,660,812	1	29,672,513	31,490,543	5.11	0.13	0.09
	42,677,946	5	30,560,109	31,567,496	3.32	-0.08	0.05
	44,889,178	5	43,784,082	44,978,312	60.16	-0.43	0.72
	45,034,833	2	45,364,686	45,589,769	12.65	-0.49	0.15
		2	45,990,820	46,476,893	159	0.48	0.62
**Chr.6**	141,858	6	34,418,280	34,746,716	6.42	0.09	0.07
		7	18,134,127	19,125,603	4.08	0.06	0.07
		7	20,031,936	21,336,059	4.61	0.11	0.12
		7	31,154,026	31,147,307	6.5	0.21	0.27
		1	31,969,210	32,182,170	4.23	0.05	0.05
		3	49,253,062	49,587,887	4.34	0.06	0.07
	367,935	7	25,827,092	32,165,223	3.44	0.04	0.05
		7	32,165,223	27,304,791	3.67	0.04	0.06
		7	27,678,256	29,578,496	5.31	0.07	0.09
		7	28,446,893	30,560,109	3.16	0.07	0.09
		8	8,967,829	10,197,480	3.63	-0.04	0.06
	519,613	4	2,582,156	7,753,676	3.15	0.04	0.05
	609,210	9	15,651,385	18,576,632	33.49	-0.56	0.58
	1,113,515	6	28,321,789	29,740,841	3.43	-0.11	0.05
		8	8,501,984	8,967,829	3	-0.05	0.05

		8	27,691,137	28,916,268	3.02	0.07	0.05
		8	30,565,945	32,092,898	4.24	0.06	0.06
	1,614,462	6	869,172	1,238,859	4.65	-0.06	0.07
		8	30,311,623	31,498,529	5.28	0.15	0.09
		8	32,092,898	32,882,885	3.89	-0.16	0.08
	1,855,586	9	42,296,922	42,740,059	43.9	-0.48	0.65
		9	52,759,823	54,043,426	68.28	-0.48	0.65
		9	54,197,831	55,084,603	72.89	-0.47	0.65
	2,471,603	6	32,943,589	33,050,487	3.02	-0.12	0.05
	2,323,221	6	32,604,905	32,989,101	8	-0.16	0.15
	2,581,120	6	32,943,589	32,989,101	4.09	0.12	0.06
		7	31,325,781	31,898,696	5	-0.12	0.08
		7	20,161,820	21,063,298	17.82	0.46	0.28
		8	40,291,590	40,513,591	12.75	0.43	0.28
		8	2,249,714	2,972,878	4.14	0.27	0.16
	2,754,693	6	29,740,841	31,154,712	3.48	0.08	0.06
		6	32,573,787	33,112,442	12.97	0.3	0.28
		6	34,420,276	34,746,716	16.79	-0.25	0.24
	2,754,709	4	37,209,479	37,285,523	4.4	-0.04	0.07
		4	38,383,279	38,711,133	6.83	0.06	0.12
	2,926,276	7	31,154,026	31,147,307	249.3	-0.49	0.66
		8	1,907,189	3,624,136	248.76	-0.5	0.66
	2,934,924	8	1,907,189	2,249,714	173.48	-0.49	0.71
	3,337,584	8	1,907,189	3,624,136	20.06	0.46	0.25
		9	40,471,848	41,216,640	129.43	0.49	0.5

	3,396,831	3	43,254,274	46,168,027	3.99	0.05	0.07
		3	46,970,660	47,358,386	4.46	0.05	0.08
		4	37,149,506	37,285,523	4.4	-0.14	0.13
		5	30,110,023	30,560,109	3.15	-0.04	0.05
	3,438,144	7	19,480,559	20,081,018	114.81	-0.46	0.69
		7	20,081,018	21,757,983	11.69	0.49	0.21
		7	30,796,174	31,147,307	116.23	-0.5	0.74
		8	1,907,189	2,249,714	32.33	-0.47	0.41
		8	2,249,714	3,438,310	71.14	-0.41	0.55
		8	35,570,051	36,874,175	11.03	-0.23	0.24
	3,632,581	6	28,001,000	29,740,841	3.45	-0.07	0.05
		6	32,604,905	32,989,101	9.07	-0.11	0.13
		6	34,420,276	34,746,716	5.23	-0.09	0.07
		7	19,480,559	21,757,983	14.55	-0.23	0.36
		7	30,796,174	31,147,307	14.1	-0.17	0.22
		8	1,907,189	3,431,963	9.3	-0.14	0.13
		8	3,840,799	4,277,590	12.54	-0.15	0.17
		8	7,085,610	8,501,984	17.95	-0.21	0.26
	4,233,358	8	38,284,837	40,261,059	14.1	0.12	0.22
	4,330,835	6	29,740,841	31,050,799	7.8	-0.15	0.19
		7	25,521,444	28,812,910	250.17	0.5	0.78
	4,337,457	6	28,307,479	31,154,712	12.58	-0.17	0.25
		6	32,057,389	32,465,403	22.6	0.31	0.35
		1	31,871,825	32,015,957	70.79	0.49	0.74
	4,568,657	6	29,740,841	32,604,905	5.09	-0.12	0.09

		8	36,714,694	37,049,093	7.04	-0.1	0.11
		8	37,896,389	38,766,407	4.59	-0.07	0.08
	4,795,485	2	39,846,687	40,239,933	6.53	-0.18	0.27
		2	41,586,441	42,150,027	4.52	0.18	0.1
		2	42,781,558	45,364,686	6.92	0.13	0.14
		9	45,631,606	47,434,928	23.74	0.42	0.49
		9	52,759,823	54,197,810	16.05	0.42	0.48
		9	54,197,810	55,084,603	18.64	0.42	0.48
		9	55,084,603	56,221,686	31.42	0.42	0.49
	4,965,763	4	884,325	1,251,708	4.28	0.13	0.06
		4	1,856,864	1,998,993	4.22	-0.06	0.06
	5,275,614	9	42,738,860	42,942,707	9.49	-0.19	0.23
		9	43,659,622	43,975,048	6.13	0.32	0.1
	5,575,891	9	40,471,848	42,942,707	35.94	0.49	0.47
		9	43,659,622	48,540,183	186.44	-0.53	0.76
		9	52,759,823	54,043,426	151.612	-0.52	0.49
		9	54,197,831	55,084,603	152.18	-0.5	0.51
	6,138,816	9	47,380,798	48,540,183	145.56	-0.47	0.93
		9	52,759,823	54,043,426	136.79	-0.47	0.96
		9	54,197,810	55,084,603	135.92	-0.47	0.96
		9	55,084,603	56,221,686	141.18	-0.47	0.96
	6,968,024	9	45,631,606	47,380,798	16	-0.44	0.33
		9	51,236,110	55,084,603	8.07	-0.24	0.26
		9	55,084,603	56,221,686	16.7	0.48	0.31
	7,288,520	3	49,009,434	49,168,131	5.91	-0.12	0.1

	7,303,794	8	45,092,476	46,168,027	5.08	0.05	0.02
		1	29,672,513	30,520,499	4.4	0.06	0.08
	7,426,409	6	33,112,437	33,632,058	9.5	0.12	0.2
		6	33,632,117	34,544,886	4.82	0.1	0.15
		6	34,746,716	35,499,378	2.97	0.06	0.07
		8	37,033,482	37,493,996	3.47	0.05	0.08
		1	29,672,530	31,597,485	14.23	0.14	0.24
	11,346,206	6	29,740,841	31,384,390	5.8	-0.14	0.16
		1	29,672,513	31,597,485	4.59	0.19	0.2
		1	31,871,825	32,182,170	41.62	0.49	0.69
	21,979,560	8	31,124,148	31,498,529	3.17	-0.07	0.08
	21,997,111	8	34,424,753	35,223,980	3.77	0.04	0.08
	21,739,348	6	31,124,408	31,972,241	21.41	0.47	0.34
		6	31,956,712	32,604,905	9.65	0.46	0.17
	29,468,820	6	28,307,479	32,465,403	31.57	-0.48	0.64
		6	34,731,063	34,986,079	34.16	-0.5	0.41
		1	31,962,655	32,182,170	47.28	0.48	0.6
	30,478,890	7	33,120,715	34,811,079	3.1	0.12	0.06
	31,087,603	2	37,565,049	37,732,654	3.4	0.13	0.06
		2	39,154,474	39,846,687	3.81	0.15	0.08
	31,678,315	5	13,395,803	22,098,390	8.8	0.12	0.23
	32,009,630	5	7,698,529	8,008,145	41.24	0.21	0.23
	32,260,632	2	32,071,506	36,548,425	4.01	-0.47	0.06
	32,994,825	9	54,043,426	55,084,603	70.78	-0.47	0.45
	33,164,527	7	26,950,787	28,812,910	78.1	0.84	0.34

		7	34,235,172	34,649,194	107.68	0.84	0.76
		8	3,624,136	3,951,788	100.86	-0.51	0.72
	33,426,574	6	33,221,090	33,632,117	3.56	0.11	0.11
		9	14,616,159	18,576,632	4.5	-0.09	0.09
	33,653,931	2	45,364,686	46,476,893	45.85	0.5	0.49
	33,823,512	7	30,305,947	30,888,816	3.51	0.1	0.06
		7	31,147,307	32,749,614	4.86	0.12	0.09
		8	6,133,733	7,960,498	4.62	-0.19	0.14
	34,116,961	8	11,153,920	25,709,306	8.35	0.18	0.15
	34,305,041	8	27,436,058	28,502,455	25.14	0.35	0.16
		8	28,446,735	29,019,358	12.18	0.19	0.09
	34,482,581	7	20,161,820	21,063,298	4.81	-0.14	0.06
		7	30,888,816	31,147,307	8.11	-0.13	0.05
		8	2,173,481	3,666,110	5.43	-0.13	0.05
		8	3,863,879	4,338,832	3.95	-0.07	0.02
		8	13,414,472	25,820,332	3.41	0.08	0.07
		8	35,223,980	37,033,482	3.08	-0.06	0.02
	34,866,397	8	28,446,735	30,311,623	37.66	-0.36	0.54
	34,866,427	4	38,711,133	39,449,540	8.3	0.09	0.16
	35,073,398	1	39,404,706	40,224,233	4.65	0.1	0.08
	35,208,168	6	28,001,000	29,740,841	5.3	-0.08	0.08
	35,394,974	9	55,084,603	56,221,686	14.23	0.48	0.49
	35,510,800	8	30,252,712	30,604,908	3.34	0.05	0.06
	35,622,907	1	24,430,388	26,421,404	8.98	0.5	0.44
		2	5,712,615	6,625,925	45.45	-0.46	0.63

		2	7,034,059	8,799,281	40.34	-0.48	0.63
		2	9,020,579	10,369,701	27.9	-0.45	0.63
		2	10,369,701	11,962,855	34.67	-0.5	0.63
	35,639,568	8	30,565,945	31,046,182	5.41	-0.17	0.24
	35,863,866	8	30,604,908	31,498,529	236	-0.52	0.92
		8	33,223,552	33,540,021	15.02	0.26	0.3
		8	33,907,057	35,570,051	94.4	0.5	0.84
**Chr.7**	610,012	1	31,969,210	32,286,203	13.48	0.23	0.16
		1	32,486,833	33,350,955	9.83	0.23	0.21
		2	467,805	693,449	6.52	-0.15	0.18
	1,237,607	8	30,565,945	30,604,908	3.06	0.04	0.05
		8	37,896,389	38,639,815	3.64	-0.05	0.06
	2,742,533	1	31,969,210	32,286,203	13.74	0.26	0.22
		1	32,500,855	33,350,955	29.52	0.48	0.5
	1,970,843	1	31,969,210	32,286,203	13.39	0.21	0.21
	2,281,477	1	31,969,210	32,286,203	4	0.11	0.04
		1	33,207,066	33,350,955	4.68	0.14	0.06
	2,459,688	2	9,020,579	11,279,783	4.67	-0.15	0.15
	2,920,547	1	32,500,855	33,207,066	65.25	0.5	0.41
	3,843,052	2	467,805	658,112	3.27	-0.07	0.05
	4,776,185	1	32,486,833	33,350,955	57.79	0.5	0.36
	7,137,577	1	2,654,669	4,067,797	30.75	-0.48	0.27
		1	4,067,797	5,009,557	33.14	-0.49	0.26
		1	6,626,055	7,717,146	27.7	0.47	0.5
		7	2,012,871	14,750,555	56.04	-0.51	0.56

	5,618,755	8	10,197,480	21,179,869	174.28	-0.49	0.87
		8	15,590,360	25,537,442	190	-0.51	0.84
		8	25,820,332	27,827,260	97.38	-0.47	0.84
		8	28,502,455	29,019,358	31.59	0.44	0.46
	11,946,406	1	31,969,210	32,257,837	3.78	0.1	0.05
	12,236,287	2	467,805	582,923	10.07	-0.14	0.09
	8,897,182	6	33,632,058	33,973,839	7.31	-0.1	0.2
		6	34,986,138	35,970,802	4.79	0.06	0.08
	14,865,543	5	35,477,616	38,308,328	3.32	-0.12	0.17
		6	283,715	495,609	52	-0.29	0.32
	14,860,658	5	44,826,206	44,846,088	6.55	0.23	0.09
		6	283,715	495,609	67	-0.49	0.61
	16,420,823	2	1,441,762	2,013,485	10	-0.18	0.18
	17,617,611	2	30,005,148	32,071,506	87	0.49	0.77
		4	37,149,407	37,770,349	4.16	-0.04	0.1
		4	37,770,349	38,358,161	3.77	-0.05	0.1
	17,749,600	5	43,784,082	44,944,140	56	-0.5	0.6
	17,765,217	6	34,746,716	34,986,079	80.69	0.49	0.72
	17,766,794	6	34,746,716	34,986,079	85.81	0.49	0.8
	18,457,345	7	20,031,936	20,161,820	6.22	0.24	0.12
		8	1,907,189	2,972,878	9.95	-0.5	0.59
	18,671,280	3	21,501,837	22,541,256	3.72	0.23	0.11
	19,169,489	3	17,693,733	18,603,221	3.16	0.12	0.08
		3	20,050,828	21,148,824	2.81	0.12	0.07
	19,360,391	5	13,395,803	18,411,778	6.23	-0.05	0.1

	19,756,247	3	18,694,440	18,830,392	3.11	-0.09	0.05
		6	7,411,505	7,650,019	3.43	-0.09	0.06
	20,056,468	3	49,030,117	49,258,598	7.47	0.21	0.22
	21,530,649	4	2,093,701	2,582,156	3.43	0.06	0.06
	21,547,021	4	34,180,137	34,526,599	2.93	-0.06	0.03
		3	49,508,996	49,798,216	4.02	0.05	0.07
	22,228,191	3	48,454,818	48,682,720	3.31	-0.08	0.06
	23,112,450	4	37,209,479	37,285,523	4.66	0.08	0.08
		4	37,610,411	38,358,161	3.4	0.08	0.06
	24,405,948	3	6,409,016	6,741,112	3.07	-0.17	0.05
		3	15,133,043	17,738,825	5.55	0.26	0.09
	29,252,490	3	14,827,022	15,091,423	3.24	-0.06	0.05
	29,534,668	4	37,765,503	38,383,223	3.23	-0.04	0.06
		4	39,659,594	40,035,645	8.81	0.14	0.26
	29,536,886	7	26,950,787	28,812,910	3.01	0.09	0.16
		7	29,858,047	30,305,947	5.32	0.1	0.15
	29,718,053	8	8,244,844	8,967,829	8.11	-0.19	0.14
		8	25,563,189	25,820,332	3.64	-0.22	0.06
		8	27,436,058	28,916,268	4.66	0.15	0.08
	30,482,984	1	40,345,474	40,708,831	10.23	-0.12	0.16
	30,534,678	8	4,146,658	6,937,176	3.55	0.04	0.1
		8	34,424,753	35,570,051	4.32	0.48	0.93
	30,644,529	8	6,937,176	7,992,779	44	0.48	0.67
	30,671,301	3	47,608,042	48,086,097	19.61	-0.47	0.25
	31,612,210	4	34,180,120	35,520,126	5.53	0.21	0.1

		4	37,139,099	37,149,407	4.09	-0.18	0.09
	31,947,427	5	26,271,809	26,552,245	3.34	-0.05	0.05
	32,087,363	5	42,003,845	43,712,480	120.32	-0.44	0.75
	32,460,960	2	11,962,855	16,148,907	3.6	-0.09	0.06
	33,263,888	1	10,090,990	10,893,951	3.41	-0.02	0.06
	33,422,959	2	23,709,262	27,583,917	9.92	0.16	0.16
	34,233,477	5	7,698,529	8,027,467	98.8	-0.39	0.67
	34,440,452	1	39,404,706	40,224,233	3.84	-0.08	0.06
**Chr.8**	1,739,577	5	35,477,616	38,308,328	19.95	-0.47	0.56
		5	39,176,254	40,145,371	17.78	-0.46	0.52
		8	27,691,137	28,502,455	37.91	-0.47	0.63
	1,777,484	4	37,285,523	37,765,503	7.58	-0.19	0.28
		5	30,858,286	33,120,715	3.49	-0.05	0.05
	2,611,296	2	37,112,626	37,732,654	3.94	-0.04	0.07
	3,240,427	7	21,335,925	21,757,983	4.25	-0.11	0.23
		8	2,249,714	2,888,331	7	-0.11	0.22
	3,531,223	3	47,608,081	48,086,069	38.87	0.33	0.59
		9	55,084,603	56,221,686	72.8	-0.47	0.65
	3,753,454	3	24,743,581	26,688,202	5.19	0.07	0.08
	3,852,339	9	33,819,413	36,540,173	126.9	-0.48	0.84
	3,907,388	6	34,544,886	34,746,716	4.2	0.06	0.07
	4,308,211	3	48,810,960	49,009,434	9.7	0.13	0.16
	4,773,472	3	15,133,043	17,728,285	3.83	-0.03	0.04
	4,521,037	3	49,168,131	49,258,598	3.84	0.06	0.05
	4,926,926	4	34,180,120	34,716,025	5.46	0.1	0.08

		4	34,716,025	35,520,126	5.57	0.11	0.08
		4	36,258,192	37,149,407	5.97	-0.11	0.09
		3	49,009,434	49,168,131	7.08	-0.1	0.09
	5,267,362	3	7,354,805	8,742,470	71.16	-0.45	0.74
	7,011,390	4	2,093,701	2,582,156	126.24	-0.47	0.87
	7,143,708	3	48,888,169	49,171,672	3.47	0.09	0.07
	7,143,730	7	11,938,862	4,056,987	31.51	0.36	0.55
		7	1,399,884	2,012,871	46.54	0.37	0.59
		7	14,970,865	14,750,555	56.22	-0.43	0.66
	7,671,664	4	34,180,120	34,716,025	8.48	0.11	0.12
		4	34,716,025	35,520,126	9.02	0.11	0.12
	8,373,414	3	48,810,960	49,009,434	4.79	0.09	0.08
	8,651,953	2	30,207,202	36,384,580	3.55	-0.08	0.06
		1	40,345,453	40,751,038	5.98	-0.1	0.1
	9,044,014	9	55,084,603	56,221,686	78.67	0.46	0.5
	9,444,121	3	49,009,381	49,168,131	6.18	-0.1	0.12
	10,675,700	7	26,950,787	28,812,910	20.77	0.26	0.47
		9	42,942,707	43,431,496	4.72	-0.15	0.11
	11,480,076	3	49,009,434	49,171,672	6.44	-0.06	0.1
	12,342,316	3	3,836,611	4,374,226	236	-0.51	0.74
	17,297,170	4	37,149,407	37,765,503	4.19	-0.1	0.08
	17,794,309	3	48,086,069	48,200,157	55.22	0.28	0.65
	26,628,195	2	26,273,973	27,678,791	140.39	-0.49	0.84
	28,527,799	7	26,950,787	28,812,910	20.01	0.44	0.54
	28,527,801	2	36,548,425	37,732,654	3.61	0.05	0.06

	28,913,468	3	17,728,285	18,694,440	3.84	0.06	0.06
	30,282,197	9	51,156,786	54,197,810	158	-0.58	0.84
	30,995,483	3	43,125,875	43,338,589	2.97	0.06	0.05
		3	45,920,744	46,580,262	4.63	-0.13	0.16
		3	46,952,491	48,086,069	4.97	-0.08	0.09
	32,325,045	3	14,514,370	15,091,423	6.35	-0.09	0.1
		3	27,168,150	39,297,616	3.3	0.06	0.06
	34,194,630	2	30,005,148	30,767,544	126	0.5	0.78
		2	36,384,580	36,548,425	9.2	-0.19	0.18
	34,300,644	5	34,502,794	34,811,079	4.35	0.14	0.16
		5	34,943,574	39,176,254	61.13	-0.5	0.69
	35,266,336	6	29,302,019	32,604,905	17.29	-0.49	0.33
		7	1,283,283	3,067,597	56.04	0.5	0.74
		7	14,970,865	16,150,646	53.32	0.5	0.62
		7	17,535,386	17,895,275	50.76	0.5	0.6
		7	19,125,603	20,031,936	10.41	-0.49	0.21
	35,632,918	3	26,538,579	39,297,616	3.78	-0.07	0.07
	35,676,551	2	46,492,383	47,140,195	94.65	-0.42	0.71
	36,695,002	2	42,584,490	42,781,558	57	-0.5	0.71
		2	1,653,133	2,013,485	7.04	-0.2	0.29
	36,695,031	7	19,480,559	20,080,988	249.92	-0.51	0.81
		9	40,248,082	40,939,008	189.6	-0.45	0.79
	36,882,442	6	31,384,390	31,972,241	36.93	0.48	0.57
	37,153,795	6	32,730,083	32,943,589	4.82	-0.1	0.08
	37,353,187	1	29,672,530	32,182,170	17.45	0.46	0.44

	37,661,656	6	34,746,716	35,160,718	5.84	0.17	0.28
		6	35,290,571	35,522,223	7.26	0.16	0.26
		7	11,938,862	3,618,028	4.54	-0.09	0.08
	37,736,721	4	33,516,646	34,324,523	79.55	0.49	0.75
	37,754,421	3	49,030,117	49,171,672	3.98	0.11	0.09
	37,766,032	9	15,651,385	18,576,632	28	-0.45	0.54
	37,955,836	7	26,950,787	28,812,910	39.44	0.48	0.61
	37,955,898	1	5,009,557	6,625,936	3.34	0.04	0.06
		1	7,349,135	9,653,120	3.49	0.04	0.06
	38,233,564	5	9,913,514	11,092,224	85.9	-0.48	0.78
		5	11,092,224	13,395,803	67.81	-0.5	0.78
	38,375,799	8	2,972,878	3,438,310	5.87	-0.21	0.36
		8	3,863,879	4,146,658	4.31	-0.1	0.11
	38,498,029	3	7,325,313	7,686,927	5.58	-0.11	0.23
	38,703,102	2	36,384,580	36,548,425	99.72	0.47	0.73
	38,938,234	7	33,501,487	34,235,172	4.19	0.12	0.07
		7	34,231,771	34,649,194	4.94	0.12	0.07
		3	48,454,818	48,682,720	3.33	0.03	0.06
	40,296,405	1	31,962,655	32,182,170	2.49	0.04	0.02
	40,454,058	6	33,112,442	33,632,058	4.31	0.08	0.1
		6	33,675,746	34,731,063	9.28	0.12	0.2
		6	34,746,716	35,160,718	9.57	0.15	0.27
		7	26,950,787	28,812,910	4.02	-0.05	0.05
		7	30,592,996	30,796,174	9.82	0.12	0.13
		7	31,898,696	32,780,901	12.45	-0.11	0.26

**Chr.9**	15,000,883	3	49,171,672	49,253,062	8.45	0.12	0.18
	15,518,496	3	49,171,672	49,253,062	5.71	0.09	0.12
	15,740,948	3	49,171,672	49,253,062	6.06	0.1	0.12
		3	49,253,062	49,587,887	6.97	0.11	0.13
	17,114,008	3	49,171,672	49,750,352	5.29	0.11	0.09
	18,795,577	3	49,171,672	49,253,062	4.92	0.08	0.11
		3	49,253,062	49,587,887	6.92	0.1	0.14
	20,602,672	3	49,253,062	49,798,216	5.4	0.07	0.11
	26,214,206	3	49,171,672	49,253,062	3.8	0.06	0.08
		3	49,253,062	49,587,864	3.47	0.06	0.08
	29,136,908	3	49,171,672	49,253,062	3.27	0.05	0.07
		3	49,253,062	49,798,216	7.89	0.07	0.14
	34,863,283	3	49,171,672	49,253,062	4.09	0.07	0.09
		3	49,253,062	49,798,216	7.36	0.09	0.15
	35,967,878	3	49,483,305	49,798,216	3.58	0.07	0.06
	36,795,823	3	49,171,672	49,258,598	3.55	0.08	0.08
		3	49,253,062	49,798,216	10.65	0.12	0.18
	38,394,110	7	34,231,711	34,649,194	30.98	0.48	0.43
		3	49,483,305	49,798,216	3.34	0.07	0.06
	40,360,009	3	49,750,352	49,918,131	5.13	0.09	0.09
	41,325,722	9	16,096,289	18,576,632	151.96	0.48	0.64
	41,612,535	3	49,171,672	49,258,598	3.6	0.06	0.07
	42,043,594	3	49,918,131	50,229,560	3.57	0.06	0.06
	42,518,489	3	49,918,131	50,229,560	3.57	0.07	0.06
	43,396,314	4	38,806,496	39,440,701	3.9	0.04	0.07

		4	39,440,701	39,449,540	5.18	0.04	0.09
	43,458,708	4	38,817,105	39,440,701	3.67	0.08	0.1
	44,566,945	4	32,859,103	34,180,137	4.94	-0.11	0.08
	44,902,703	9	47,380,798	49,055,032	146.33	-0.46	0.91
		9	52,759,823	54,043,426	136.38	-0.48	0.95
		9	54,197,810	55,084,603	135.71	-0.47	0.95
		9	55,084,603	56,221,686	140.69	-0.48	0.95
	46,485,080	7	28,812,910	30,592,996	3.6	-0.14	0.06
		7	31,147,307	32,749,614	5.48	0.18	0.09
		7	32,849,878	33,409,621	7.23	0.15	0.07
	46,506,202	3	50,229,560	50,282,134	3.53	-0.06	0.05
	47,987,555	5	7,698,529	8,008,145	26.02	-0.45	0.49
	49,940,941	9	45,631,606	47,380,798	13.33	0.31	0.43
		9	54,197,810	55,084,603	24.22	0.47	0.58
		9	55,084,603	56,221,686	50.19	0.48	0.65
	51,465,877	2	41,586,441	42,465,031	3.44	-0.06	0.05
		8	3,863,939	4,146,658	3.03	0.05	0.05
	54,120,618	1	6,625,936	7,717,146	31.27	-0.41	0.53
	54,120,666	9	43,659,622	43,975,058	85.8	-0.43	0.84
	56,099,023	2	24,428,975	27,678,791	61.15	-0.49	0.74
		2	29,841,451	30,207,202	7.05	0.29	0.19

Table S2

Table S2 Segregation distortion (P<0.05) of SNPs detected in this trial

Chromosomes	Positions (bp)	*P* value
**Chr.1**	3,801,842	4.18722E-07
	3,801,858	2.7014E-06
	3,801,889	4.18876E-06
	3,801,893	5.35E-06
	3,801,897	8.65135E-06
	4,067,797	0.042267425
	4,214,268	0.024968253
	4,807,833	0.019089769
	5,009,557	0.004847096
	6,625,936	0.044760936
	6,625,942	0.044760936
	6,626,040	0.044760936
	6,888,874	1.48302E-07
	6,913,226	0.017692197
	7,654,059	0.030971435
	7,668,290	0.035342083
	7,681,038	0.010197877
	7,717,163	0.026165409
	7,717,179	0.007290358
	9,895,522	0.022167908
	10,021,619	2.895E-05
	10,203,267	0.002421467
	10,203,337	0.001862846
	10,400,534	0.032053594
	10,519,087	0.006250477
	10,519,091	0.004090967
	10,519,106	0.0201152
	10,519,126	0.005171111
	10,519,127	0.007819219
	10,519,133	0.005171111
	10,519,202	0.004965342
	10,710,214	0.000152873
	10,798,005	0.036190038
	10,893,882	0.002281937
	11,521,883	0.029274259
	12,532,582	0.037043385
	16,457,381	1.10889E-07
	21,853,358	0.007955439
	24,524,546	3.08937E-06

	24,524,599	6.26114E-05
	24,524,604	6.26114E-05
	27,521,305	2.46333E-07
	30,499,670	6.86107E-05
	30,499,698	2.50979E-08
	30,499,701	0.000916024
	30,499,789	2.88449E-05
	30,512,625	0.000494598
	30,512,627	0.001748637
	30,512,637	0.008404419
	30,512,666	0.004446738
	30,621,350	0.007819219
	30,792,892	0.047425393
	31,797,736	0.047953134
	32,182,248	0.029523219
	32,257,837	0.033006262
	32,317,295	0.029763655
	33,350,939	0.002421467
	33,350,942	0.002421467
	33,350,948	0.003125579
	33,350,955	0.003125579
	33,350,985	0.003125579
	34,929,774	0.032007673
	36,776,922	0.013122334
	36,776,934	0.005417667
	36,777,031	0.032124575
	40,345,474	0.014029063
**Chr.2**	402,257	0.006493165
	402,262	0.003164962
	402,267	0.008385492
	402,268	0.008385492
	402,270	0.012643832
	402,274	0.012643832
	402,356	0.020550304
	402,368	0.034790145
	589,280	0.032007673
	1,081,925	1.10889E-07
	1,081,978	0.016156932
	1,082,017	0.033286683
	1,082,020	0.039634097
	1,194,195	0.016949739
	1,201,962	5.66661E-06
	1,261,329	0.044065407

	1,261,987	0.045945095
	1,262,040	0.047135637
	1,262,048	0.047135637
	1,262,093	0.009673851
	1,350,774	0.047953134
	1,350,785	0.043114452
	1,441,762	0.000126337
	1,639,697	0.010487006
	1,639,788	0.017503607
	1,639,796	0.01430588
	1,653,064	0.021889521
	1,653,112	0.037901955
	1,653,133	0.044065407
	1,653,161	0.045020887
	1,979,238	0.045020887
	2,013,485	0.024340924
	5,748,644	0.018715356
	5,922,737	0.029205514
	6,625,925	0.000439587
	6,625,955	0.000439587
	6,654,498	1.35568E-06
	6,714,680	0.002860811
	6,714,696	0.00370901
	6,744,260	0.000925274
	6,744,286	0.000241546
	6,860,949	0.002420151
	6,876,527	0.000170953
	6,923,524	0.000159675
	6,923,531	0.000159675
	6,952,358	0.00031436
	6,984,903	0.001701872
	6,990,452	0.00333036
	6,992,069	0.000113274
	7,020,810	0.000675471
	7,034,050	4.004E-14
	7,034,059	1.20823E-14
	7,034,085	7.89642E-15
	7,034,112	3.36391E-15
	7,511,317	0.000512112
	7,610,609	1.19333E-05
	7,610,624	1.19333E-05
	7,610,649	1.41921E-05
	7,610,652	1.41921E-05

	7,610,668	1.41921E-05
	7,610,676	1.85754E-05
	7,610,711	6.83433E-09
	7,755,690	2.25015E-05
	7,779,774	1.71627E-05
	7,779,830	1.88236E-05
	7,825,022	6.91472E-05
	7,825,236	0.001209846
	7,825,241	0.000688514
	7,825,244	0.000688514
	7,832,981	5.05506E-05
	7,833,007	4.69125E-05
	7,833,009	7.6125E-05
	7,837,170	2.64794E-05
	7,837,229	2.64794E-05
	7,837,239	2.64794E-05
	7,837,272	2.64794E-05
	7,837,273	2.64794E-05
	7,837,317	1.60181E-05
	8,098,413	5.0909E-16
	8,098,428	1.3633E-13
	8,098,446	1.82242E-13
	8,098,476	3.1997E-13
	8,098,500	6.20264E-13
	8,103,456	4.31204E-06
	8,108,165	7.42076E-05
	8,108,236	7.42076E-05
	8,108,237	7.42076E-05
	8,108,241	7.42076E-05
	8,108,263	9.39657E-05
	8,241,597	4.27134E-05
	8,281,956	4.0113E-07
	8,281,963	6.3561E-07
	8,281,967	6.3561E-07
	8,760,425	0.00092365
	8,760,459	0.00092365
	8,760,466	0.00092365
	8,760,514	0.00092365
	8,799,281	5.78099E-05
	9,020,508	0.000973971
	9,020,509	0.001643731
	9,020,516	0.002180459
	9,020,579	0.000207502

	9,020,590	0.000161635
	9,020,619	0.000125592
	9,110,373	0.000402244
	9,110,417	0.000104714
	9,616,244	6.31779E-05
	9,656,385	5.55416E-05
	9,656,401	5.55416E-05
	9,656,406	9.66587E-05
	9,656,420	7.6125E-05
	9,656,813	8.02864E-06
	9,656,828	9.53384E-06
	9,657,368	1.0219E-05
	9,657,496	1.41921E-05
	9,658,844	3.39672E-05
	9,658,845	3.39672E-05
	9,658,875	7.33298E-06
	9,658,923	4.52451E-14
	9,658,944	3.39672E-05
	9,665,046	1.73724E-05
	9,665,159	1.59925E-05
	9,724,272	6.86107E-05
	9,757,061	5.62898E-06
	9,859,962	5.98236E-05
	9,897,706	4.00416E-06
	9,897,734	1.48276E-06
	9,953,710	0.000568402
	9,953,723	0.000386488
	10,167,354	0.002311375
	10,321,901	0.001501976
	10,354,943	2.27826E-06
	10,359,631	2.41359E-08
	10,359,670	6.75311E-09
	10,359,672	1.50886E-08
	10,359,703	4.11326E-09
	10,364,858	3.9623E-05
	10,364,881	6.76142E-06
	10,364,926	1.73724E-05
	10,369,701	1.1038E-08
	10,369,782	4.01161E-09
	11,279,783	2.07339E-08
	11,841,420	1.25155E-10
	11,844,032	1.24186E-12
	11,888,506	1.6373E-09

	11,888,508	1.6373E-09
	11,888,530	2.34594E-09
	11,888,548	3.51413E-10
	11,962,855	6.409E-09
	11,962,861	6.409E-09
	11,962,955	6.409E-09
	11,962,969	1.06062E-10
	11,962,979	6.409E-09
	12,018,737	2.13284E-11
	12,018,761	2.13284E-11
	12,028,100	1.96125E-08
	12,082,506	1.45577E-10
	12,114,264	1.53367E-07
	12,330,228	6.8575E-10
	12,349,445	7.04649E-11
	12,349,477	1.72286E-11
	13,753,764	1.69229E-10
	13,804,542	9.27708E-13
	13,804,560	1.66286E-12
	13,804,575	4.22519E-12
	13,804,589	4.22519E-12
	13,804,602	1.66286E-12
	13,804,632	1.66286E-12
	13,804,648	1.66286E-12
	13,857,860	4.80629E-10
	14,149,675	4.19404E-14
	14,215,277	7.99775E-10
	14,215,302	1.11069E-09
	14,215,308	2.13284E-11
	14,225,108	1.36588E-13
	14,277,712	3.37184E-10
	14,279,575	2.69059E-15
	14,279,592	3.78593E-13
	14,279,627	1.94085E-12
	14,283,847	7.13806E-11
	14,283,928	5.42989E-10
	14,313,504	6.31497E-09
	14,313,512	2.86661E-10
	14,624,049	7.93835E-08
	14,936,820	1.05573E-11
	14,961,591	6.37715E-12
	14,993,639	1.03196E-16
	14,993,640	1.50151E-11

	15,042,659	8.92317E-12
	15,042,763	9.557E-11
	15,057,783	2.14152E-08
	15,065,379	4.63389E-10
	15,065,396	9.25928E-09
	15,069,311	8.83174E-11
	15,146,120	7.35226E-10
	15,156,540	2.10923E-08
	15,156,573	2.10923E-08
	15,156,580	4.14539E-08
	15,220,386	2.02414E-08
	15,249,139	7.99775E-10
	15,259,544	2.55197E-07
	15,266,316	4.1291E-10
	15,304,393	1.2894E-11
	15,304,402	1.2894E-11
	15,363,117	6.76869E-10
	15,363,148	6.26793E-11
	15,363,170	3.48028E-10
	15,400,085	9.11544E-08
	15,400,118	1.05719E-07
	15,400,139	7.43098E-07
	15,413,061	3.91766E-11
	15,413,099	2.03339E-11
	15,413,102	6.71646E-10
	15,415,538	4.64098E-08
	15,415,562	5.72307E-09
	15,445,810	2.92488E-10
	15,445,815	1.03377E-10
	15,519,455	8.78085E-09
	15,519,456	4.67064E-09
	15,550,583	1.30592E-09
	15,567,918	1.10919E-09
	15,585,669	6.80917E-07
	15,611,809	1.65462E-10
	15,655,505	1.54023E-09
	15,746,656	9.84401E-10
	15,789,029	2.81086E-10
	15,922,159	1.53163E-09
	15,940,923	2.30838E-09
	16,085,326	7.31231E-10
	16,104,658	6.53146E-09
	16,104,751	5.61149E-09

	16,104,801	3.14202E-08
	16,148,907	9.4236E-10
	16,181,488	2.59856E-11
	16,181,490	1.24601E-11
	16,181,594	1.18253E-11
	16,181,613	1.16889E-11
	16,284,436	5.75069E-10
	16,308,116	2.9173E-09
	16,357,843	1.41499E-10
	16,393,609	3.39861E-09
	16,393,690	3.39861E-09
	16,558,000	9.4236E-10
	16,558,013	1.53163E-09
	16,558,019	1.53163E-09
	16,558,032	1.53163E-09
	16,562,209	1.03421E-11
	16,562,240	1.10123E-09
	16,562,298	2.69942E-09
	16,604,448	1.54173E-08
	16,604,458	1.10806E-08
	16,604,533	7.95071E-09
	16,694,031	2.9173E-09
	16,694,044	1.53163E-09
	16,694,115	4.80629E-10
	16,720,180	3.31288E-10
	16,720,185	8.48722E-10
	16,725,887	2.11538E-09
	16,793,405	1.53163E-09
	16,793,423	2.11538E-09
	16,793,432	9.4236E-10
	16,844,609	1.47643E-09
	16,844,643	8.7191E-10
	16,844,705	7.2181E-10
	16,844,723	1.96616E-10
	16,844,744	6.60838E-11
	16,898,904	4.01719E-09
	16,898,915	7.58264E-09
	16,900,777	9.36449E-07
	16,948,462	2.04723E-10
	16,948,548	1.17059E-10
	17,726,996	4.02051E-08
	17,726,997	4.80699E-08
	18,133,290	3.62409E-06

	18,241,256	1.10919E-09
	18,500,637	7.83631E-09
	18,596,106	5.29397E-11
	20,511,452	4.01719E-09
	21,874,258	6.22822E-11
	22,340,329	1.25557E-08
	23,132,463	3.91991E-08
	23,339,523	5.02051E-07
	23,565,763	4.85699E-10
	23,597,564	3.25118E-08
	23,689,163	1.3163E-07
	23,709,262	1.80746E-09
	23,805,023	5.76493E-08
	23,890,985	1.7927E-09
	24,148,121	3.55863E-07
	24,148,207	5.72307E-09
	24,302,231	0.000846298
	24,428,975	1.83459E-07
	24,428,985	4.90861E-07
	24,640,108	2.99526E-11
	24,643,415	1.98267E-06
	24,658,265	2.15286E-07
	24,658,707	2.73233E-10
	24,678,830	3.17897E-05
	24,678,832	3.17897E-05
	24,678,871	1.13496E-06
	24,777,411	3.40043E-12
	24,777,478	8.01254E-13
	24,807,784	4.44252E-11
	24,817,878	9.41095E-08
	24,847,551	3.0819E-10
	24,847,592	1.97872E-12
	24,847,619	3.73548E-10
	24,847,621	2.63967E-10
	24,847,642	2.63967E-10
	24,925,597	2.90567E-12
	24,962,379	1.03935E-08
	24,962,431	1.88764E-08
	24,982,892	1.12642E-11
	24,989,839	1.26983E-10
	24,989,869	6.98994E-08
	24,991,594	3.49038E-08
	24,991,615	3.02575E-11

	24,991,647	1.64055E-08
	25,041,456	1.02903E-07
	25,054,742	1.03586E-10
	25,060,711	6.66409E-08
	25,105,407	5.75069E-10
	25,159,326	1.72326E-08
	25,159,385	2.41411E-10
	25,159,394	3.43892E-10
	25,176,082	1.34949E-06
	25,200,764	1.20905E-05
	25,200,781	0.00013617
	25,200,822	6.26114E-05
	25,228,324	2.30233E-08
	25,228,334	1.45602E-08
	25,228,387	3.14202E-08
	25,328,774	3.03999E-07
	25,328,782	6.7551E-07
	25,328,801	2.55817E-07
	25,338,417	5.76493E-08
	25,338,428	3.5055E-08
	25,371,795	4.66301E-11
	25,371,815	2.47607E-09
	25,371,834	2.94628E-09
	25,371,837	4.11326E-09
	25,371,857	1.07283E-09
	25,388,351	5.40301E-08
	25,388,353	8.29884E-08
	25,388,371	8.78085E-09
	25,388,431	3.49038E-08
	25,388,465	4.03192E-10
	25,388,871	1.08761E-06
	25,392,564	4.55311E-08
	25,392,572	4.55311E-08
	25,502,871	1.50246E-07
	25,540,590	5.5259E-09
	25,540,672	1.81766E-08
	25,558,719	7.93813E-10
	25,558,754	6.48773E-09
	25,585,932	7.83631E-09
	25,585,939	4.53412E-07
	25,672,351	1.03935E-08
	25,694,791	4.73463E-08
	25,694,823	2.94878E-09

	25,694,852	6.41178E-08
	25,694,857	6.41178E-08
	25,694,879	5.61704E-08
	25,694,881	2.51628E-09
	25,694,889	3.61076E-08
	25,846,555	2.55817E-07
	25,846,652	6.57194E-12
	25,846,660	1.69727E-07
	25,846,675	1.94349E-08
	25,885,440	0.002186742
	25,901,637	2.12014E-07
	25,965,383	5.13112E-06
	26,040,589	2.9173E-09
	26,040,594	4.67064E-09
	26,040,642	1.11069E-09
	26,085,185	4.70898E-09
	26,187,845	5.10287E-12
	26,273,914	5.09892E-10
	26,273,924	4.0637E-08
	26,273,973	3.43892E-10
	26,275,330	1.54799E-06
	26,275,349	6.80234E-08
	26,426,969	0.000188925
	26,426,985	0.000188925
	26,449,005	2.30057E-12
	26,510,003	2.4573E-08
	26,510,049	4.41301E-08
	26,524,285	4.22488E-07
	26,524,303	3.31077E-08
	26,524,308	3.31077E-08
	26,588,531	8.10248E-07
	26,588,550	6.79202E-05
	26,588,555	3.46711E-05
	26,588,581	0.000120015
	26,588,609	8.70756E-05
	26,600,382	3.827E-07
	26,614,213	7.76461E-07
	26,643,852	1.18859E-12
	26,643,882	3.1927E-11
	26,643,894	1.8103E-11
	26,643,895	4.55532E-11
	26,643,974	1.65462E-10
	26,643,994	6.63162E-10

	26,664,438	2.12726E-07
	26,684,843	1.82917E-07
	26,759,746	1.38363E-05
	26,759,769	6.10644E-06
	26,759,849	1.60282E-06
	26,773,911	2.19314E-10
	26,781,995	4.54945E-06
	26,811,566	2.7812E-11
	26,811,570	7.97551E-11
	26,847,280	3.46479E-09
	26,988,742	2.92467E-16
	27,001,753	5.08901E-07
	27,157,729	1.31462E-06
	27,157,805	6.7551E-07
	27,157,809	6.04905E-07
	27,200,620	9.95831E-07
	27,583,887	5.70062E-10
	27,583,917	3.26197E-09
	27,678,262	1.78837E-06
	27,678,264	1.78837E-06
	27,678,265	1.78837E-06
	27,678,791	4.08993E-07
	27,684,367	3.85962E-06
	27,690,632	4.82721E-07
	28,140,865	6.70003E-09
	28,149,321	4.51676E-07
	28,827,121	1.45774E-13
	28,827,140	1.53746E-12
	28,827,184	3.32413E-14
	28,827,236	3.0082E-06
	28,834,627	1.46671E-05
	28,834,639	2.18253E-05
	28,834,643	0.000352451
	29,474,149	2.11765E-06
	29,841,451	9.95831E-07
	30,005,148	1.56269E-06
	30,083,264	1.31462E-06
	30,136,970	3.827E-07
	30,136,985	2.55817E-07
	30,179,258	0.000171914
	30,179,319	3.17897E-05
	30,206,650	2.87287E-07
	30,207,179	2.87287E-07

	30,207,202	2.87287E-07
	30,208,472	2.87287E-07
	30,208,480	2.87287E-07
	30,208,486	2.87287E-07
	30,249,904	8.1967E-06
	30,253,980	3.21001E-06
	30,390,806	2.55817E-07
	30,767,544	1.4574E-06
	30,922,956	9.95831E-07
	31,071,277	7.30254E-08
	32,071,506	1.6047E-06
	32,197,516	1.23907E-05
	37,112,626	0.02242164
	37,414,342	0.007790879
	37,414,383	0.026992288
	37,456,801	0.026421749
	37,456,802	0.026421749
	37,456,804	0.026421749
	37,456,827	0.026421749
	37,456,836	0.014137971
	37,456,859	0.031250021
	37,482,747	0.005615999
	37,482,752	0.004489904
	37,482,795	5.5259E-09
	37,482,860	1.81084E-08
	37,482,861	0.002699796
	37,492,205	0.022493441
	37,492,437	0.000673859
	37,492,440	0.000835478
	37,492,511	0.027116031
	37,492,515	0.000673859
	37,508,762	0.001422898
	37,508,781	0.000812176
	37,565,049	0.013376338
	37,732,654	0.00698409
	37,991,019	0.014915625
	40,239,933	0.009280908
	41,586,441	0.029274259
	41,875,407	0.002396234
	43,516,636	1.71829E-10
**Chr.3**	3,682,667	0.022996412
	3,801,785	0.01760753
	3,801,796	0.014484057

	3,836,611	0.019108865
	3,945,017	0.025601131
	4,361,592	0.002631652
	4,374,226	0.039634097
	4,374,710	0.043153941
	4,374,736	0.037249173
	4,374,809	0.036446982
	4,383,534	0.024340924
	5,280,080	0.006098946
	5,292,592	0.003125579
	5,295,829	0.00074896
	5,304,938	0.00031436
	5,492,071	0.000224051
	5,492,081	9.73457E-05
	5,530,642	0.000165442
	5,530,663	1.34956E-05
	5,530,725	0.000208334
	5,536,301	6.86454E-05
	5,536,335	0.002716447
	5,643,049	1.0219E-05
	5,739,377	0.000236382
	5,752,704	0.002114711
	5,849,862	0.000222034
	5,851,369	9.66587E-05
	5,923,492	3.671E-05
	6,159,771	9.79123E-06
	6,220,007	0.029274259
	6,347,743	1.96125E-08
	6,409,016	4.88497E-08
	6,409,120	8.45861E-08
	6,409,121	1.15234E-07
	6,409,133	5.74198E-09
	6,419,793	4.41301E-08
	6,468,738	3.63801E-06
	6,470,203	3.43006E-05
	6,643,552	9.6652E-05
	6,646,458	9.53384E-06
	6,680,203	5.46593E-06
	6,680,249	8.16688E-06
	6,680,278	6.13117E-06
	6,740,761	6.01739E-08
	6,740,787	0.000224051
	6,740,835	3.06943E-05

	6,741,112	3.56421E-05
	6,776,864	3.29746E-06
	7,021,133	4.82721E-07
	7,028,371	3.20981E-07
	7,028,408	9.26957E-08
	7,066,251	4.65842E-10
	7,177,757	1.61057E-06
	7,210,960	5.37056E-08
	7,220,184	2.99728E-07
	7,325,313	6.78693E-07
	7,354,805	8.97082E-09
	7,426,559	4.09033E-10
	7,521,633	2.08009E-07
	7,551,905	1.53367E-07
	7,551,929	8.94473E-07
	7,686,912	9.4236E-10
	7,686,917	9.4236E-10
	7,686,927	9.4236E-10
	7,686,932	9.4236E-10
	7,687,000	7.76447E-13
	7,709,487	8.65136E-09
	8,128,552	3.65965E-11
	8,128,557	3.82094E-12
	8,128,559	2.55963E-12
	8,214,983	2.69613E-06
	8,305,593	9.11544E-08
	8,305,613	2.14261E-08
	8,305,627	9.11544E-08
	8,305,637	1.71689E-07
	8,324,606	1.42238E-08
	8,324,690	2.5689E-08
	8,379,630	3.46479E-09
	8,436,298	5.73303E-07
	8,493,427	2.97524E-09
	8,543,371	4.18876E-06
	8,543,372	2.27351E-06
	8,624,089	2.70591E-07
	8,639,476	3.85454E-10
	8,667,493	2.42415E-10
	8,697,677	5.92525E-06
	8,742,470	8.70499E-08
	8,750,234	8.33474E-09
	8,763,158	1.81697E-09

	8,763,203	5.64632E-10
	8,791,020	1.53163E-09
	9,057,026	1.29675E-09
	9,081,485	2.11538E-09
	9,081,560	3.39861E-09
	9,081,597	2.96062E-10
	9,718,109	9.11544E-08
	9,791,541	3.49038E-08
	9,796,477	3.44631E-09
	9,906,912	1.78356E-08
	10,089,111	6.7551E-07
	10,089,126	6.7551E-07
	10,089,201	8.29884E-08
	10,104,693	9.41095E-08
	10,153,487	4.76318E-06
	10,219,284	5.68069E-08
	10,352,303	1.52738E-06
	10,454,349	1.64055E-08
	10,492,603	3.80759E-07
	12,301,459	6.76962E-12
	12,343,012	2.44264E-05
	12,410,535	0.012986559
	13,002,234	5.43742E-05
	13,160,284	0.004022779
	13,494,321	0.011623305
	13,504,582	0.003589203
	13,541,119	0.038947459
	13,813,846	9.11802E-06
	13,830,583	0.038765578
	13,856,676	0.018026138
	13,957,365	0.044760936
	13,995,211	0.014759476
	13,995,235	0.018554894
	14,031,782	0.004965342
	14,031,850	0.00077753
	14,077,784	9.40013E-05
	14,077,852	1.70901E-06
	14,514,370	0.026239443
	14,514,379	0.026239443
	14,514,391	0.026239443
	14,514,396	0.026239443
	14,892,214	1.03062E-06
	14,892,267	1.61057E-06

	14,897,147	0.000355112
	14,897,207	0.000494598
	14,951,945	0.030625743
	14,964,445	0.001761702
	15,074,223	0.044065407
	15,091,423	0.034499692
	15,091,508	0.024412071
	15,091,513	0.014759476
	15,091,521	0.009721743
	15,112,485	0.030210963
	15,112,497	0.025078856
	15,133,036	0.024968253
	15,133,043	0.024968253
	15,133,048	0.029205514
	17,693,733	0.028365508
	17,728,285	2.38858E-05
	17,984,077	0.025696421
	17,999,489	0.021397571
	17,999,490	0.021397571
	18,084,756	0.008299216
	18,603,221	0.043153941
	18,603,309	0.043153941
	18,694,440	0.011238372
	18,823,342	0.010760155
	18,830,392	0.02242164
	18,830,411	0.02242164
	18,830,436	0.02242164
	18,830,448	0.047912763
	18,830,468	0.012602026
	18,880,555	0.010956616
	18,880,565	0.02242164
	19,443,224	0.020550304
	20,050,828	0.007790879
	22,332,846	0.017096987
	22,593,708	0.01417213
	24,743,581	0.010760155
	26,077,505	0.021210756
	26,538,571	0.000459626
	26,538,579	0.000328154
	26,722,400	0.022948311
	39,297,616	0.02128836
	39,573,013	0.026500283
	39,654,514	2.78596E-07

	39,654,604	4.13873E-08
	39,654,635	3.67258E-06
	39,654,647	4.18722E-07
	39,711,228	0.005775499
	39,711,232	0.018964764
	39,719,496	0.016446561
	39,738,534	0.002115607
	39,738,593	0.002115607
	39,738,640	0.017118241
	39,738,680	0.017118241
	39,752,695	0.006349725
	40,409,012	0.000373694
	40,409,049	0.000320979
	40,706,280	0.040805368
	40,706,319	0.029572207
	40,874,235	0.035342083
	40,948,767	0.030625743
	40,971,512	0.048884643
	40,971,621	0.008385492
	40,971,691	0.04222118
	41,019,995	0.044401268
	42,680,151	0.005088186
	42,758,582	0.027198402
	42,833,101	0.042168202
	42,839,245	2.84177E-05
	42,839,283	0.027806897
	42,839,299	0.023103397
	42,839,351	0.023103397
	42,839,352	0.027806897
	43,255,224	0.041810548
	43,338,532	0.000718536
	43,338,589	0.000718536
	43,338,624	6.16451E-05
	43,343,756	0.019630659
	43,350,398	0.01760753
	45,309,848	0.000238563
	45,309,917	0.004148197
	45,346,589	0.003983152
	45,346,590	0.003983152
	45,346,653	0.002469714
	45,346,672	0.003821977
	45,538,338	0.008351424
	45,538,476	0.016987408

	45,727,938	0.026239443
	46,970,660	9.10334E-06
	46,970,708	0.000249832
	46,970,720	2.99306E-05
	46,970,760	0.000227763
	46,970,777	0.000743854
	47,069,572	0.001422898
	47,358,359	4.10901E-05
	47,358,386	0.01219285
	49,278,950	0.048717604
	49,483,305	1.3232E-08
	50,290,247	0.020550304
**Chr.4**	1,062,117	0.041140637
	1,139,929	4.81386E-11
	1,139,933	7.58619E-12
	1,139,941	5.10287E-12
	1,251,708	0.034856853
	1,251,754	0.034856853
	1,355,950	0.003125579
	1,357,815	0.035649494
	4,013,411	0.013376338
	4,013,494	0.015795192
	4,013,500	0.015795192
	4,013,531	0.015795192
	4,013,533	0.015795192
	4,074,903	0.002942482
	4,097,756	0.009058175
	4,097,762	0.009058175
	4,125,542	0.00940718
	4,125,568	0.013560121
	4,207,253	0.008705089
	4,207,272	0.007000942
	4,238,040	0.00906008
	5,387,428	0.048332248
	5,903,151	0.027827712
	5,903,153	0.033947901
	6,456,410	0.011310672
	6,456,474	0.002699796
	7,753,651	0.001643731
	7,753,670	0.001643731
	7,753,676	0.001643731
	19,986,488	0.048332248
	23,614,287	0.033006262

	27,625,194	0.039634097
	33,516,646	0.005209294
	34,180,155	0.01255492
	34,180,174	0.018399654
	34,180,210	0.0051014
	34,324,523	1.4574E-06
	34,526,525	0.032053594
	34,716,025	0.000547862
	35,520,126	7.39299E-09
	35,763,857	0.048717604
	35,763,874	0.011616892
	37,209,479	0.044859248
	37,243,522	0.003026856
	37,243,532	0.015257513
	38,711,133	0.002979467
	40,129,637	0.029912993
	40,129,676	0.00726114
	40,129,679	0.029205514
**Chr.5**	8,785,240	0.010661056
	9,086,137	6.48773E-09
	9,205,606	0.002426238
	9,205,662	0.021210756
	9,319,126	0.003374168
	9,801,913	0.000769726
	9,801,921	0.000769726
	9,848,856	0.009950829
	9,848,920	0.00851939
	13,451,679	0.027806897
	14,014,861	0.021291758
	14,014,919	0.033286683
	14,156,791	0.00437609
	14,156,842	0.007534765
	14,156,843	0.009387054
	14,156,849	0.011623305
	14,156,881	0.014759476
	14,156,890	0.014759476
	14,167,184	0.015861334
	14,167,588	0.00726114
	14,328,665	0.015795192
	14,328,835	0.006051565
	14,335,952	0.005209294
	14,335,999	0.004148197
	14,336,042	0.007000942

	14,336,052	0.004446738
	14,343,072	0.027229654
	14,658,939	0.015795192
	14,771,789	0.012222801
	14,771,845	0.029205514
	14,789,074	0.001647561
	15,087,628	0.010956616
	15,475,901	0.006522388
	18,520,351	0.014915625
	24,100,953	0.003664669
	24,222,575	0.007000942
	24,374,189	0.008636217
	24,440,238	6.43424E-15
	24,440,239	2.5115E-13
	24,440,251	1.64959E-12
	24,441,943	0.009960646
	24,441,968	0.008351424
	24,462,960	0.004489904
	24,462,987	0.016634679
	24,534,568	0.000786722
	24,739,536	0.01255492
	24,739,544	0.014988031
	24,765,385	0.027229654
	24,771,317	3.38127E-05
	24,771,318	3.38127E-05
	24,771,345	0.016477645
	24,771,351	0.016477645
	24,771,365	0.01385854
	24,771,382	0.01385854
	24,771,388	0.01385854
	24,771,412	0.01385854
	24,774,519	0.012196685
	24,774,525	0.001761261
	24,774,533	0.019319502
	24,774,559	0.019319502
	24,774,565	0.019319502
	24,806,202	0.000504848
	24,825,697	1.09124E-13
	24,825,756	0.00698409
	25,060,949	0.005381544
	25,060,985	0.008109116
	25,071,211	0.011117562
	25,076,475	0.000134333

	25,076,504	4.3289E-06
	25,153,221	0.010956616
	25,425,054	0.001063115
	25,425,055	0.001063115
	25,610,194	0.045020887
	25,820,637	0.036446982
	25,820,642	0.010956616
	25,827,092	0.010619545
	25,857,715	0.010760155
	25,858,827	0.0400668
	25,911,505	0.04050735
	25,968,251	0.001493386
	25,968,252	0.001274996
	25,968,343	0.010619545
	25,968,347	0.010619545
	25,972,513	0.018102456
	25,972,551	0.018102456
	25,972,579	0.02128836
	25,972,604	0.009960646
	25,972,611	0.014915625
	25,976,974	4.86223E-08
	26,271,809	0.000687104
	26,271,822	0.000687104
	26,279,869	0.038433934
	26,285,486	1.71627E-05
	26,296,304	0.000938086
	26,296,322	0.000373694
	26,437,173	0.011623305
	26,437,210	0.009387054
	26,552,156	0.0340692
	26,552,236	0.033286683
	27,308,193	0.005761163
	27,349,804	0.029205514
	27,474,923	0.004446738
	27,474,937	0.005596631
	27,474,959	0.005381544
	27,678,256	0.007819219
	28,446,817	0.008636217
	28,446,893	0.008636217
	28,473,825	0.010408347
	28,473,901	0.00726114
	29,662,576	1.1831E-08
	29,662,629	0.048829999

	29,662,635	0.001862846
	29,662,637	0.001862846
	29,675,076	0.001825948
	29,675,098	0.001716502
	30,560,109	5.5892E-05
	32,165,223	0.004266725
	33,101,462	0.01148051
	33,116,694	0.041226838
	33,116,709	0.025078856
	33,116,766	0.002935887
	33,153,058	0.042267425
	33,154,493	0.005417667
	33,278,063	0.040290545
	33,611,551	0.04222118
	33,902,886	3.54286E-07
	33,902,895	6.78693E-07
	33,902,968	4.18876E-06
	34,113,836	0.022957515
	34,385,284	0.034856853
	34,385,325	0.020730059
	34,385,340	0.024340924
	34,463,136	0.035649494
	34,578,543	0.048884643
	34,681,856	0.006641886
	34,866,033	0.003025697
	34,866,035	0.000136379
	34,943,574	0.04598072
	34,983,321	0.036678688
	35,011,742	8.16656E-05
	35,092,246	1.08196E-05
	35,092,252	1.5621E-05
	35,121,509	0.001078408
	35,121,531	0.001078408
	35,143,023	0.009374769
	35,477,616	0.02128836
	38,308,328	0.006522388
	40,145,371	0.024968253
	42,996,909	0.017503607
	42,996,963	0.029205514
	43,085,651	0.045945095
	43,177,640	0.00533143
	43,311,570	0.026883071
	43,311,636	0.026883071

	43,311,639	0.026883071
	43,351,923	0.033286683
	43,352,047	0.004965342
	43,784,082	0.005031491
	44,826,164	0.029205514
	44,826,179	0.0340692
	44,826,206	0.019089769
	44,834,216	0.042267425
	44,846,088	0.001114781
	44,944,140	0.013376338
	44,978,312	0.010956616
	45,012,605	0.020177457
	45,012,781	0.013554668
	45,012,784	0.013554668
	45,056,882	0.002935887
**Chr.6**	543,648	0.006522388
	674,824	0.04550027
	1,238,859	0.042413064
	1,496,370	3.80396E-08
	1,720,562	1.34374E-05
	1,720,596	0.022270982
	1,737,910	0.000497304
	1,737,916	0.000940681
	2,471,403	0.024353122
	2,818,492	2.18656E-06
	2,818,500	5.26186E-05
	2,818,526	0.001672622
	2,818,557	0.000971152
	2,818,566	0.000406952
	2,980,330	0.017692197
	3,034,049	0.004677735
	3,034,052	0.019931201
	3,362,659	0.022957515
	3,445,153	0.001209846
	4,408,241	0.002436735
	4,408,286	0.000210354
	4,421,530	2.99306E-05
	5,841,126	0.040290545
	6,492,952	0.037514009
	7,776,508	0.0400947
	7,776,550	0.021210756
	7,776,586	0.01417213
	7,776,587	0.017692197

	13,151,753	0.049534623
	21,509,339	1.25878E-06
	21,560,355	3.21596E-05
	21,573,867	0.001663872
	21,573,871	0.000292126
	21,573,892	0.000150163
	27,299,873	0.030753565
	27,299,908	0.025696421
	27,327,757	0.045885804
	27,579,642	0.019089769
	27,604,609	0.000549255
	27,639,071	0.010287539
	27,639,135	0.004022779
	27,644,997	0.004489904
	27,740,761	0.010956616
	27,895,607	0.038947459
	27,982,984	0.005031491
	28,001,000	0.007483071
	28,307,468	0.004847096
	28,307,479	0.004847096
	28,307,500	0.00698409
	28,307,503	0.00698409
	28,307,542	0.046917168
	28,321,676	0.022493441
	28,377,081	0.002265825
	28,377,090	0.002265825
	28,377,095	0.002265825
	28,399,255	0.000879956
	28,399,336	0.000594713
	28,425,953	0.004446738
	28,457,960	0.002265825
	29,192,943	0.034808484
	29,290,064	0.024340924
	29,290,128	0.029205514
	29,290,131	0.028503437
	29,290,135	0.026430976
	29,290,142	0.016156932
	29,290,143	0.013637726
	29,290,171	0.009011089
	29,301,928	0.045020887
	29,302,019	0.045020887
	29,354,180	0.02657629
	29,739,234	0.030753565

	31,050,799	0.003374168
	32,485,966	0.046140154
	32,604,905	0.000834408
	32,730,083	0.000834408
	32,730,096	0.000488208
	32,989,101	0.004847096
	32,989,105	0.004847096
	33,046,062	0.004666565
	33,112,437	0.002527347
	33,112,442	0.002527347
	33,112,525	0.002942482
	33,120,696	0.001362545
	33,120,759	0.002575051
	33,973,839	0.000606767
	34,042,120	0.001209846
	34,418,280	0.029763655
	34,420,276	0.010381796
	34,544,937	0.047953134
	34,544,951	0.039359513
	34,986,079	0.00185041
	34,986,138	0.000879454
	34,986,178	0.000938086
	35,160,718	0.013554668
	35,255,619	0.030971435
	35,290,571	0.018602931
	35,499,378	0.007534765
	35,522,223	0.003562965
	35,671,386	0.000900936
	35,698,024	0.036888432
	35,711,449	0.004489904
	35,723,520	0.000163751
	35,723,553	9.61659E-05
	35,723,591	9.61659E-05
	35,818,996	0.005171111
	35,819,061	0.010062191
	35,831,867	0.000928533
	35,831,895	0.007000942
	35,831,896	0.007000942
**Chr.7**	330,917	0.046944734
	1,086,666	2.59982E-05
	1,388,493	0.012196685
	1,388,494	0.01515844
	1,388,553	0.000481026

	1,399,831	0.045945095
	1,399,884	0.016462314
	1,399,886	0.016462314
	1,399,892	0.000137892
	1,607,300	4.18876E-06
	1,760,555	0.023751661
	1,760,562	0.013417543
	1,760,602	0.015974425
	1,831,175	0.048993504
	1,862,921	0.001643731
	1,935,178	0.000210354
	2,142,270	0.033286683
	2,142,329	0.028503437
	2,142,357	0.028503437
	2,189,431	0.001761261
	2,549,983	0.01255492
	2,550,010	0.018964764
	2,550,018	0.01255492
	2,550,030	0.015477852
	2,550,060	0.01255492
	2,550,116	0.000606767
	2,550,118	0.000606767
	2,550,127	0.001017241
	2,550,132	1.2923E-05
	2,707,369	0.037249173
	2,707,402	0.037249173
	2,707,413	0.026239443
	2,707,435	0.031343632
	2,773,487	0.030192048
	2,773,498	0.009280908
	2,773,584	0.008719498
	2,807,403	0.007290358
	2,807,414	0.009950829
	2,849,229	0.021397571
	2,888,596	0.005683389
	2,888,676	0.026992288
	2,888,682	0.009374769
	2,888,712	0.007423454
	2,906,396	0.026992288
	2,906,419	0.01090041
	2,906,482	0.005683389
	3,038,287	2.55197E-07
	4,595,408	0.019800726

	4,675,217	0.002288632
	5,170,450	0.024412071
	5,261,380	0.000669729
	6,166,222	0.001862846
	6,917,581	4.42252E-05
	6,917,585	0.04222118
	7,521,283	0.002511666
	7,980,393	0.032007673
	8,098,358	0.044760936
	8,098,461	0.009374769
	9,020,260	0.006655606
	9,211,277	0.030753565
	10,223,268	0.028707871
	11,862,623	0.048829999
	12,512,999	0.00108799
	13,233,558	0.00940718
	13,461,745	0.020501076
	14,760,227	0.000971152
	14,760,251	0.000219473
	14,760,255	0.000219473
	14,760,270	5.35805E-07
	15,615,844	9.60685E-07
	15,615,845	6.86109E-06
	15,615,857	4.88458E-05
	16,483,176	0.010074807
	16,483,183	0.03299679
	16,483,203	0.029523219
	16,483,214	0.017530324
	16,483,231	0.039806094
	16,580,793	0.016446561
	16,580,852	2.32894E-05
	16,581,360	0.008711913
	16,593,643	0.017757835
	16,593,660	0.000671888
	16,593,759	0.002755077
	16,607,222	0.001088217
	16,607,231	0.001088217
	16,607,257	0.001728331
	16,607,305	0.001408456
	16,607,335	0.001088217
	16,607,336	0.002214298
	16,607,343	0.001408456
	16,607,356	0.001408456

	16,663,418	0.007000942
	16,691,000	0.002307489
	16,801,779	0.031753344
	17,443,073	2.84177E-05
	17,443,095	2.84177E-05
	17,443,117	6.76962E-12
	17,443,120	1.43242E-08
	17,559,823	4.53898E-06
	17,559,831	2.26752E-05
	17,559,906	0.000224051
	17,675,369	0.009673851
	17,675,399	0.014710445
	17,860,887	0.018399654
	17,895,275	0.022996412
	18,133,435	0.00418122
	18,134,035	0.001811585
	18,134,127	0.006741391
	18,134,160	0.008071488
	18,431,370	0.016243043
	18,431,429	0.016243043
	18,440,344	0.000115617
	18,448,609	0.004965342
	18,448,689	0.026430976
	18,483,320	0.001546204
	18,483,343	0.002180459
	18,566,663	0.000208334
	18,566,665	0.000208334
	18,566,800	1.12161E-05
	18,893,951	0.004666565
	19,125,603	0.024826784
	19,449,663	0.000203778
	19,454,395	0.001209846
	19,480,470	0.004886988
	19,480,501	0.005320964
	19,480,506	0.006982318
	19,480,514	0.029456388
	19,480,520	0.048993504
	19,480,531	0.000916024
	19,507,373	2.18253E-05
	19,507,378	2.7014E-06
	19,507,408	1.49328E-06
	21,012,550	0.047912763
	21,336,019	0.028503437

	23,281,887	0.009760742
	23,334,572	0.001959645
	29,858,047	0.02534732
	29,858,168	0.00280161
	30,409,257	0.013554668
	30,409,270	0.02657629
	30,409,271	0.02657629
	30,409,293	0.018428887
	32,749,614	0.014484057
	32,849,878	0.006435092
	32,882,766	0.014915625
	32,882,772	0.014915625
	33,001,711	0.026500283
	33,120,971	2.91957E-05
	33,121,045	2.91957E-05
	33,329,520	0.00185041
	33,344,431	0.000410355
	33,406,731	0.001625152
	33,409,621	0.025078856
	33,409,633	0.029456388
	33,490,647	0.00027038
	33,500,019	0.018399654
	33,501,487	0.000124236
	33,501,523	6.86454E-05
	33,513,940	0.041140637
	33,513,971	0.015219242
	33,514,025	0.015219242
	33,524,782	0.002575051
	34,231,685	5.1774E-05
	34,231,711	0.000879454
	34,231,752	0.001179784
	34,231,771	0.000512112
	34,231,782	0.000938086
	34,235,172	0.008058115
	34,235,173	0.009747808
**Chr.8**	1,315,959	0.008058115
	3,069,653	2.94557E-09
	3,069,667	9.72232E-06
	3,069,670	9.72232E-06
	3,069,745	0.000207502
	3,069,764	0.000207502
	3,069,782	3.06943E-05
	3,948,750	1.11636E-07

	3,948,768	1.83972E-06
	3,948,772	1.83972E-06
	4,281,826	0.041810548
	4,281,855	0.013098344
	4,281,875	0.026500283
	4,281,932	0.032832308
	4,338,824	0.023751661
	4,338,825	0.023751661
	4,338,827	0.023751661
	4,338,832	0.001143177
	5,577,905	0.038999957
	6,133,674	8.93338E-05
	6,826,114	0.046917168
	7,166,169	0.006367308
	7,166,171	0.0025662
	7,222,370	0.000546293
	7,222,415	0.000178129
	7,432,471	0.044401268
	7,432,510	0.023738519
	7,717,203	0.000938086
	8,504,481	1.32131E-09
	8,967,820	0.003761823
	8,967,828	0.008711913
	8,967,829	0.011134105
	10,594,221	0.005545668
	10,594,261	0.005545668
	10,929,744	0.011134105
	11,321,045	0.000547862
	11,808,765	0.009387054
	12,249,530	0.041140637
	12,291,346	1.11636E-07
	12,680,222	0.032509449
	13,518,176	0.000537097
	13,641,388	2.42847E-06
	16,556,830	0.044760936
	18,031,864	0.004080991
	25,209,443	0.0400947
	25,209,488	0.0400947
	25,209,489	0.0400947
	25,563,130	0.000115617
	25,563,179	0.002190094
	25,563,187	0.000364932
	25,563,189	0.002860811

	27,828,365	0.035342083
	27,828,402	4.53898E-06
	28,520,909	0.0340692
	28,520,990	0.029205514
	28,531,517	0.018554894
	28,531,591	0.019630659
	28,531,601	0.019630659
	28,601,158	0.000823349
	28,608,839	0.021852255
	28,608,864	3.55994E-10
	28,608,867	4.78026E-14
	28,608,868	9.13619E-12
	28,709,891	5.15615E-15
	28,790,326	0.000980383
	28,790,345	0.001274996
	28,790,356	0.001274996
	28,790,357	0.001274996
	28,790,362	0.001274996
	28,790,395	0.001274996
	28,831,874	0.008351424
	28,831,967	0.008351424
	28,831,971	0.001274996
	28,831,995	0.008351424
	28,832,005	0.00018873
	28,832,007	0.000341747
	28,832,008	0.000341747
	28,914,789	0.029912993
	28,914,837	0.029912993
	28,914,857	0.029912993
	28,914,865	0.029912993
	28,914,892	0.029912993
	28,914,923	0.029912993
	28,916,196	0.030625743
	28,916,197	0.030625743
	28,916,268	0.030625743
	28,916,300	0.030625743
	29,019,358	0.024826784
	29,019,383	0.029763655
	29,238,896	5.46593E-06
	29,267,018	0.018428887
	29,281,172	0.024968253
	29,284,363	0.030210963
	29,284,428	0.030210963

	29,284,433	0.035342083
	29,360,291	0.001510414
	29,371,085	0.041385177
	29,927,156	0.030625743
	29,957,956	0.020700276
	29,957,991	0.020700276
	29,957,993	0.041226838
	29,958,024	0.00671439
	29,958,026	0.011238372
	29,958,037	0.011238372
	29,958,039	0.009058175
	29,958,055	0.009058175
	29,958,097	0.002172154
	29,958,113	0.00011472
	30,054,955	0.045885804
	30,206,436	0.000916024
	30,206,443	8.84342E-06
	30,206,452	8.84342E-06
	30,206,490	0.000863043
	32,361,656	0.027806897
	32,394,813	0.000525557
	32,394,814	0.005417667
	32,394,827	0.000718536
	32,394,829	0.000565077
	32,394,855	0.000381058
	32,394,869	0.00851939
	32,394,920	0.008150972
	32,394,925	0.000623256
	32,438,312	0.046944734
	32,438,314	0.033286683
	32,438,341	0.002755077
	32,557,170	0.001926604
	32,557,192	0.002469714
	32,592,155	0.023719265
	32,694,840	0.000497304
	32,694,850	0.000275865
	32,737,432	0.010287539
	32,737,504	0.014915625
	32,737,550	0.010287539
	32,738,837	0.012411331
	32,877,104	0.003215884
	33,068,128	0.018102456
	33,068,198	0.029912993

	33,082,602	0.046604896
	33,109,387	0.00161134
	33,379,630	0.025696421
	33,508,288	4.34887E-05
	33,508,339	1.49023E-05
	33,508,356	5.31213E-05
	33,529,825	3.44875E-05
	33,529,893	0.001862846
	33,540,021	0.001078987
	33,540,038	0.000568531
	33,663,014	0.005031491
	33,663,039	0.005031491
	33,663,073	0.005031491
	33,663,091	0.003197874
	33,734,212	0.016377309
	33,734,213	0.016377309
	33,762,705	0.027466086
	33,905,688	5.31213E-05
	33,907,032	0.005458784
	33,907,057	0.002979467
	33,996,474	0.027229654
	34,042,368	0.016209542
	34,042,441	0.038812958
	34,054,949	0.037901955
	34,176,535	0.001152045
	34,207,044	0.034499692
	34,236,312	0.017530324
	34,236,370	0.040805368
	34,287,410	0.046944734
	34,400,732	0.032066531
	34,400,819	0.037249173
	34,404,588	0.002699796
	34,404,692	0.007439182
	34,431,159	0.002699796
	34,431,178	0.026883071
	34,431,193	0.026883071
	35,379,813	0.01674627
	35,379,819	0.001503251
	35,379,905	4.42252E-05
	35,449,997	8.39566E-07
	35,450,065	1.71829E-10
	35,450,073	3.14656E-12
	35,450,074	3.14656E-12

	36,782,728	0.033947901
	37,645,228	0.04222118
	38,002,886	0.047425393
	38,015,407	2.5535E-07
	38,879,247	0.012982974
	38,997,206	0.039806094
	40,291,590	0.002180459
	40,291,591	0.002180459
**Chr.9**	14,616,159	9.8533E-08
	15,385,607	8.08536E-17
	15,651,385	2.49767E-09
	16,096,289	5.65855E-15
	16,295,929	2.52138E-15
	18,576,632	2.38654E-12
	18,689,607	1.83002E-11
	18,848,482	3.51787E-16
	18,853,030	1.50429E-10
	18,884,363	1.15727E-12
	18,901,540	2.36614E-16
	18,901,546	2.36614E-16
	18,901,547	2.36614E-16
	18,908,456	2.19678E-11
	18,911,316	2.36614E-16
	18,911,342	2.36614E-16
	18,911,403	1.12364E-09
	19,006,545	3.72827E-15
	19,018,518	2.06236E-12
	19,018,585	1.78936E-14
	19,025,932	1.11636E-07
	19,025,975	4.86223E-08
	19,077,515	5.59711E-13
	19,077,516	7.01864E-12
	19,106,768	5.49382E-13
	19,106,813	2.55963E-12
	19,106,855	1.94905E-11
	19,161,343	2.42415E-10
	19,195,558	1.1252E-12
	19,195,575	6.12821E-13
	19,195,605	1.1252E-12
	19,195,656	9.14435E-14
	19,216,600	1.68963E-13
	19,216,667	1.54089E-12
	19,216,694	1.16443E-13

	19,218,969	1.16443E-13
	19,227,743	4.80699E-08
	19,313,072	7.6054E-12
	19,319,497	1.89853E-09
	19,420,739	1.33266E-13
	19,493,680	4.32072E-14
	19,493,713	2.30712E-14
	19,570,948	3.59533E-16
	19,594,151	1.2894E-11
	19,635,200	6.49543E-16
	19,635,212	5.83058E-16
	19,638,969	7.41372E-12
	19,639,032	3.92787E-17
	19,646,868	5.23039E-17
	19,646,934	7.14832E-17
	19,646,954	1.87444E-18
	19,654,569	1.77042E-16
	19,687,275	7.58619E-12
	19,754,217	1.96011E-10
	19,765,131	3.0049E-11
	19,869,347	1.54089E-12
	19,869,349	2.75999E-12
	19,869,395	7.00481E-12
	19,869,396	2.75999E-12
	19,885,935	1.07623E-15
	19,885,973	2.89331E-11
	19,940,618	4.22519E-12
	19,982,390	5.35864E-17
	20,020,865	1.30543E-08
	20,020,894	1.81766E-08
	20,020,919	2.52659E-08
	20,020,921	1.10166E-08
	20,020,960	1.27715E-07
	20,039,437	1.54089E-12
	20,039,466	3.96018E-12
	20,119,797	5.37265E-16
	20,119,827	4.35812E-14
	20,119,869	8.1888E-14
	20,119,918	1.93575E-13
	20,119,923	1.93575E-13
	20,119,927	1.93575E-13
	20,127,095	5.67541E-12
	20,272,074	2.95368E-12

	20,272,131	9.27708E-13
	20,306,893	7.7863E-16
	20,518,761	2.38654E-12
	20,816,318	6.43538E-13
	21,291,430	1.66286E-12
	21,611,452	3.24655E-16
	21,657,484	4.71098E-14
	21,721,749	6.76745E-13
	21,755,974	2.16071E-07
	21,809,849	5.02372E-13
	21,822,397	2.77178E-15
	21,901,159	1.1962E-10
	21,902,785	1.16638E-12
	22,109,285	5.15615E-15
	22,157,396	7.04649E-11
	22,157,413	2.50886E-10
	22,157,485	1.9148E-10
	22,157,490	3.72841E-11
	22,172,351	9.40301E-15
	22,206,845	2.63357E-16
	22,206,861	1.62736E-18
	22,206,907	1.56272E-14
	22,303,798	8.01627E-14
	22,303,915	4.32072E-14
	22,312,501	1.66984E-10
	22,481,788	1.62736E-18
	22,497,974	3.75132E-13
	22,526,997	3.54286E-07
	22,543,006	2.38471E-11
	22,866,549	2.48277E-11
	22,866,557	3.54286E-07
	22,866,561	7.87068E-13
	22,866,572	1.80055E-11
	22,866,599	1.76594E-08
	22,930,856	2.87068E-11
	22,945,193	8.52272E-13
	22,945,234	1.99053E-10
	23,161,779	6.9319E-16
	23,161,796	6.43538E-13
	23,180,126	5.52151E-15
	23,372,196	1.2053E-13
	23,461,689	7.94273E-17
	23,612,761	4.87695E-14

	23,612,763	4.87695E-14
	23,648,877	0.000876479
	23,768,647	1.60184E-15
	23,827,549	2.40042E-14
	23,957,200	8.10493E-15
	23,957,299	9.66403E-16
	24,135,465	6.03678E-12
	24,167,932	2.60671E-15
	24,168,060	1.16889E-11
	24,178,081	1.28809E-10
	24,184,390	9.97025E-14
	24,184,452	3.52832E-16
	24,202,888	9.66403E-16
	24,277,136	1.81084E-08
	24,290,217	1.96177E-16
	24,366,260	7.13676E-09
	24,454,404	4.56123E-12
	24,594,164	4.11579E-09
	24,594,230	2.88514E-09
	24,612,629	2.13341E-15
	24,612,651	1.19844E-14
	24,709,290	8.95443E-10
	24,715,351	1.43831E-12
	24,858,796	2.344E-16
	24,869,138	2.83597E-14
	25,067,847	7.92049E-12
	25,119,209	6.03678E-12
	25,141,176	9.46429E-17
	25,185,052	1.38175E-14
	25,185,056	1.70357E-15
	25,190,086	5.37265E-16
	25,401,287	3.24655E-16
	25,523,249	1.27983E-17
	25,557,657	8.1079E-14
	25,557,697	4.29186E-13
	25,557,725	1.53746E-12
	25,813,196	7.14236E-13
	25,813,287	5.00091E-16
	25,909,673	1.8103E-11
	25,909,765	3.01828E-14
	26,077,915	3.29212E-10
	26,080,028	5.37679E-12
	26,157,331	7.22607E-14

	26,325,235	3.69142E-14
	26,325,289	3.2635E-10
	26,325,327	1.09777E-13
	26,368,892	4.88758E-08
	26,419,708	5.42989E-10
	26,598,302	5.50092E-14
	26,640,178	1.23947E-13
	26,766,137	2.24188E-13
	26,766,189	1.68886E-11
	26,766,260	8.15149E-12
	26,777,911	2.26142E-13
	26,877,421	4.8983E-12
	26,981,441	7.75541E-16
	27,076,245	5.7743E-17
	27,168,626	8.10493E-15
	27,168,639	2.50979E-08
	27,168,672	2.26142E-13
	27,321,894	4.94611E-14
	27,804,766	1.29617E-13
	27,804,792	3.24406E-11
	27,817,722	1.56272E-14
	27,953,169	5.31754E-17
	28,019,953	2.95368E-12
	28,020,023	1.1854E-16
	28,193,162	7.21303E-15
	28,210,827	5.00252E-11
	28,356,276	8.29284E-11
	28,382,204	2.95368E-12
	28,476,828	1.69464E-15
	28,476,839	1.99053E-10
	28,554,381	1.52247E-12
	28,572,924	2.22481E-11
	28,573,018	5.42289E-16
	28,650,939	5.70062E-10
	28,651,002	1.6688E-16
	28,684,795	9.94749E-19
	28,684,868	6.43538E-13
	28,804,490	1.66286E-12
	28,845,222	1.69875E-16
	28,864,525	8.29284E-11
	29,005,704	2.62202E-17
	29,129,561	3.63838E-15
	29,251,063	1.12212E-13

	29,356,556	4.66301E-11
	29,422,436	9.66359E-19
	29,434,697	2.69059E-15
	29,434,745	1.80141E-14
	29,434,747	1.20951E-14
	29,448,018	2.06236E-12
	29,529,036	9.44443E-17
	29,596,165	5.91947E-15
	29,627,133	1.80141E-14
	29,627,156	1.20951E-14
	29,627,178	2.96757E-15
	29,680,463	5.74126E-11
	29,923,890	8.90064E-13
	30,046,266	7.49036E-12
	30,147,150	2.52138E-15
	30,192,092	3.1813E-14
	30,192,115	1.3276E-16
	30,192,121	5.81846E-16
	30,470,194	5.31754E-17
	30,470,214	1.43831E-12
	30,470,272	1.43831E-12
	30,470,278	1.43831E-12
	30,470,284	4.67636E-16
	30,485,985	6.20953E-12
	30,702,456	2.21578E-13
	30,702,457	1.01751E-17
	30,702,484	2.21578E-13
	30,711,017	5.0909E-16
	30,724,973	1.15371E-14
	30,725,093	8.1079E-14
	30,745,963	1.45573E-10
	30,746,042	3.32413E-14
	31,189,779	6.48881E-17
	31,279,394	1.6464E-11
	31,284,856	1.66286E-12
	31,331,896	3.74535E-11
	31,331,943	7.7496E-11
	31,331,945	1.11247E-10
	31,339,289	3.29404E-12
	31,393,323	9.12177E-19
	31,393,406	1.82303E-17
	31,486,334	7.08423E-11
	31,853,987	6.94874E-15

	31,854,057	6.94874E-15
	31,886,140	2.72361E-09
	31,886,178	3.53209E-09
	31,939,337	1.6582E-11
	31,964,053	8.22509E-14
	31,964,103	1.55092E-13
	32,015,819	6.68595E-13
	32,015,830	1.47081E-12
	32,015,848	3.69142E-14
	32,016,903	7.40865E-13
	32,016,915	7.40865E-13
	32,016,920	4.07083E-13
	32,017,006	1.06907E-12
	32,054,347	3.19285E-10
	32,077,126	5.31754E-17
	32,077,133	1.3119E-18
	32,088,871	5.2256E-16
	32,088,945	5.2256E-16
	32,088,960	7.75541E-16
	32,088,990	5.2256E-16
	32,089,438	5.37265E-16
	32,089,514	5.94699E-16
	32,089,524	1.6287E-12
	32,089,545	1.98182E-15
	32,147,949	6.22964E-17
	32,147,969	2.24366E-14
	32,158,196	6.03678E-12
	32,158,227	8.61459E-12
	32,158,236	8.61459E-12
	32,158,276	8.61459E-12
	32,158,309	3.96018E-12
	32,179,332	7.04748E-11
	32,210,518	3.73503E-13
	32,210,532	1.89784E-12
	32,341,207	1.11247E-10
	32,341,230	2.55963E-12
	32,341,239	2.55963E-12
	32,341,256	1.17002E-11
	32,375,959	1.80141E-14
	32,375,975	1.80141E-14
	32,376,007	3.32413E-14
	32,376,038	8.05893E-12
	32,416,105	1.73664E-12

	32,625,476	2.1099E-17
	32,633,135	9.2366E-13
	32,633,191	9.00467E-15
	32,636,876	1.01362E-13
	32,636,883	4.02367E-13
	32,636,887	6.29584E-10
	32,727,966	2.73341E-14
	32,797,215	5.2256E-16
	32,859,504	5.10587E-10
	32,859,552	3.39456E-13
	32,948,761	9.83641E-16
	33,164,064	1.33478E-16
	33,164,076	8.7556E-16
	33,164,143	4.38504E-16
	33,164,159	5.94699E-16
	33,164,173	3.97436E-16
	33,174,257	6.37039E-10
	33,211,676	1.09124E-13
	33,211,688	8.92317E-12
	33,211,711	1.31894E-11
	33,211,718	1.31894E-11
	33,243,511	3.1654E-15
	33,392,170	2.82716E-15
	33,392,220	4.69218E-15
	33,486,375	5.87928E-17
	33,486,416	2.36614E-16
	33,486,434	4.67636E-16
	33,486,468	4.67636E-16
	33,486,471	4.67636E-16
	33,486,478	1.43831E-12
	33,486,516	4.67636E-16
	33,569,686	3.52161E-17
	33,672,220	5.58596E-13
	33,710,252	2.84137E-12
	33,710,340	1.31894E-11
	33,710,360	9.97025E-14
	33,756,016	7.94273E-17
	33,819,351	1.20951E-14
	33,819,370	1.80141E-14
	33,819,413	4.22494E-15
	33,819,439	2.99526E-11
	33,833,110	1.79952E-18
	33,833,174	5.31754E-17

	33,833,200	1.1854E-16
	33,833,219	1.66286E-12
	33,870,946	2.40042E-14
	33,870,970	4.09822E-12
	33,871,020	7.37927E-14
	33,893,918	1.542E-12
	33,893,931	9.72863E-09
	33,912,262	1.19699E-15
	34,107,328	1.97737E-15
	34,107,380	1.33478E-16
	34,107,385	6.32425E-16
	34,107,401	1.16555E-14
	34,107,403	2.85793E-12
	34,187,535	3.90101E-09
	34,187,538	1.19104E-13
	34,257,227	5.87928E-17
	34,377,720	1.92278E-17
	34,377,732	1.66286E-12
	34,377,743	2.44906E-13
	34,377,829	2.40387E-16
	34,377,830	1.52383E-13
	34,459,871	1.3633E-13
	34,459,872	2.70939E-13
	34,459,883	4.09108E-14
	34,459,922	1.65128E-14
	34,465,985	9.22326E-19
	34,479,899	5.65692E-18
	34,479,973	8.51208E-18
	34,508,474	2.84112E-11
	34,532,199	2.50352E-17
	34,532,203	2.56285E-16
	34,532,205	2.50352E-17
	34,532,282	3.25221E-17
	34,669,577	1.49141E-14
	34,676,029	3.51413E-10
	34,676,077	6.73744E-10
	34,757,374	1.19655E-15
	34,844,239	1.4708E-14
	34,884,816	2.1099E-17
	35,047,283	7.88987E-11
	35,211,950	1.68963E-13
	35,266,696	2.06707E-15
	35,266,700	3.16507E-15

	35,277,122	0.000194911
	35,386,009	3.03116E-19
	35,395,496	6.70243E-17
	35,395,497	2.01058E-16
	35,395,519	1.89358E-17
	35,395,566	1.58155E-15
	35,395,579	1.04877E-15
	35,395,598	3.3724E-13
	35,395,599	1.64959E-12
	36,540,173	1.29196E-18
	36,540,178	2.95624E-18
	38,196,068	2.85549E-09
	40,248,044	2.62118E-14
	40,248,045	2.62118E-14
	40,248,047	1.38175E-14
	40,248,082	2.62118E-14
	40,248,123	2.53404E-13
	40,248,148	3.83578E-14
	40,248,151	3.83578E-14
	40,248,171	3.83578E-14
	40,471,848	1.16889E-11
	40,642,957	9.27708E-13
	40,697,539	2.0049E-13
	40,939,008	9.6014E-11
	40,939,043	6.94806E-10
	41,066,079	2.38654E-12
	41,175,491	9.79669E-13
	41,212,068	5.65103E-08
	41,212,100	7.92821E-08
	41,216,640	3.96018E-12
	41,216,643	1.92125E-12
	41,216,697	4.07083E-13
	41,373,737	2.77738E-08
	41,409,270	4.90861E-07
	41,434,805	2.53541E-07
	41,491,622	1.00288E-07
	41,790,266	5.25964E-09
	42,296,922	3.44631E-09
	42,715,806	3.99095E-08
	42,715,864	8.29884E-08
	42,738,860	3.66279E-09
	42,738,897	4.53136E-09
	42,740,056	2.01641E-07

	42,740,059	1.57632E-07
	42,798,814	6.1521E-08
	42,798,860	6.1521E-08
	42,832,759	4.29186E-13
	42,832,768	1.5203E-13
	42,832,803	2.16088E-13
	42,899,643	5.08901E-07
	42,918,784	4.80834E-09
	42,918,815	9.13163E-09
	42,942,707	5.40301E-08
	43,286,118	4.65664E-06
	43,358,147	0.000212183
	43,361,132	2.77509E-06
	43,361,202	2.77509E-06
	43,361,416	0.002057885
	43,361,453	0.000249832
	43,373,242	1.31462E-06
	43,431,380	1.78837E-06
	43,431,434	2.05906E-06
	43,431,496	1.91574E-16
	43,434,731	4.10453E-05
	43,485,982	2.97554E-08
	43,659,622	1.34829E-05
	43,913,898	4.9817E-06
	43,913,925	3.67258E-06
	43,975,048	0.002395101
	43,975,058	0.002281937
	44,320,789	0.000488208
	44,320,796	0.000369931
	44,342,424	9.08392E-07
	44,342,425	9.08392E-07
	44,342,439	9.08392E-07
	44,342,465	4.01953E-07
	44,342,485	2.64165E-07
	44,342,493	2.64165E-07
	44,342,518	8.7191E-10
	44,342,544	1.6464E-11
	45,002,711	0.000732096
	45,002,726	8.20422E-05
	45,158,832	0.000415418
	45,158,855	0.000415418
	45,425,960	0.000516317
	45,426,057	0.000545539

	45,462,710	0.000711811
	45,462,712	0.000711811
	45,462,755	0.000606304
	45,462,775	0.000606304
	45,462,799	0.000606304
	45,462,800	0.000606304
	45,631,606	0.008925834
	47,338,555	0.021592197
	51,156,786	0.042168202
	54,197,810	0.032509449
	54,197,831	0.027806897
	54,197,907	0.008705089
